# Supplementary material for: Formulation of substrates with agricultural and forestry wastes for Camellia oleifera Abel seedling cultivation
Source: PLoS One. 2022 Jul 18;17(7):e0265979. doi: 10.1371/journal.pone.0265979 (PMC9292103; doi:10.1371/journal.pone.0265979)
Supplement: Attachment — Submitted filename: PONE-D-22-07125_R1.pdf [file pone.0265979.s003.pdf]

PLOS ONE

Formulation of substrates with agricultural and forestry wastes for Camellia oleifera Abel seedling cultivation

--Manuscript Draft--

**Manuscript Number:**	PONE-D-22-07125R1
**Article Type:**	Research Article
**Full Title:**	Formulation of substrates with agricultural and forestry wastes for Camellia oleifera Abel seedling cultivation
**Short Title:**	Substrate breeding of agricultural and forestry wastes
**Corresponding Author:**	Jinping Zhang<br>Research Institute of Subtropical Forestry Chinese Academy of Forestry<br>Hangzhou, CHINA
**Keywords:**	Cultivation substrate; compound substrate; agricultural and forestry waste; camellia oleifera; physical and chemical properties
**Abstract:**	Abstracts<br><br>Five compound substrates for the cultivation of Camellia oleifera Abel seedlings were obtained by substituting the peats in conventional substrate formulas with the composts of Camellia oleifera shell, pine chips, palm fiber residues, chicken manure, and sheep manure. The changes in the physical and chemical properties of the substrate after seedling cultivation were determined and the effects of these substrates on the development of Camellia oleifera seedling were analyzed. It was found that the survival rates of the one-year-old seedlings produced from stem cuttings in all groups were greater than 97.5% after 6 months cultivation. The seedlings exhibited greater seedling heights, larger ground diameters, longer root systems and larger total root volume than those obtained with conventional substrates. The compound substrate formulated with the compost of Camellia oleifera shell+ palm fiber residue+ chicken manure (A3), vermiculite and perlite (6:3:1) is the most optimal, which gives the 100% survival rate of cultivated seedlings, the greatest seedling heights, and the largest ground diameters. In particular, the ground diameters and 26.67% of the seedling heights reach the grade 1 standard for the two-year-old seedlings.
**Order of Authors:**	Fei Zhou
	Nianjin Wang
	Jinping Zhang
	Xiaohua Yao
	Tiantian Zhang
	Xiaofeng Zhang
	Lingyan Zhan
	Jieman Li
**Response to Reviewers:**	I have read your reply to the manuscript and modified it according to your opinions.<br>The following are the replies one by one.<br>The whole manuscript has been numbered.<br>The format of the references has been revised to meet plos ONE requirements.<br>The grammar of the whole article has been checked and revised in detail, including "we" and "the paper or study....".<br>The format of tables and figures in the article was also modified, and the tables and pictures were all put at the end of the manuscript. Only "Figure 1" and "Table 1" were left in the position of the charts in the text for illustration.<br>Table 2 has been reflected in the manuscript.<br>The new Funding Statement was attached to the cover letter.
**Additional Information:**	

Powered by Editorial Manager® and ProduXion Manager® from Aries Systems Corporation

Question	Response
**Financial Disclosure**<br><br>Enter a financial disclosure statement that describes the sources of funding for the work included in this submission. Review the submission guidelines for detailed requirements. View published research articles from *PLOS ONE* for specific examples.<br><br>This statement is required for submission and **will appear in the published article** if the submission is accepted. Please make sure it is accurate.<br><br>**Unfunded studies**<br>Enter: *The author(s) received no specific funding for this work.*<br><br>**Funded studies**<br>Enter a statement with the following details:<br>• Initials of the authors who received each award<br>• Grant numbers awarded to each author<br>• The full name of each funder<br>• URL of each funder website<br>• Did the sponsors or funders play any role in the study design, data collection and analysis, decision to publish, or preparation of the manuscript?<br>• **NO** - Include this sentence at the end of your statement: *The funders had no role in study design, data collection and analysis, decision to publish, or preparation of the manuscript.*<br>• **YES** - Specify the role(s) played.<br><br>* typeset	The funders had no role in study design, data collection and analysis, decision to publish, or preparation of the manuscript.
**Competing Interests**<br><br>Use the instructions below to enter a competing interest statement for this submission. On behalf of all authors, disclose any competing interests that could be perceived to bias this work—acknowledging all financial support and any other relevant financial or non-financial competing interests.	The authors have declared that no competing interests exist.

This statement is required for submission and **will appear in the published article** if the submission is accepted. Please make sure it is accurate and that any funding sources listed in your Funding Information later in the submission form are also declared in your Financial Disclosure statement.<br><br>View published research articles from *PLOS ONE* for specific examples.<br><br>**NO authors have competing interests**<br><br>Enter: *The authors have declared that no competing interests exist.*<br><br>**Authors with competing interests**<br><br>Enter competing interest details beginning with this statement:<br><br>*I have read the journal's policy and the authors of this manuscript have the following competing interests: [insert competing interests here]*<br><br>* typeset	
**Ethics Statement**<br><br>Enter an ethics statement for this submission. This statement is required if the study involved:<br><br>• Human participants<br>• Human specimens or tissue<br>• Vertebrate animals or cephalopods<br>• Vertebrate embryos or tissues<br>• Field research<br><br>Write "N/A" if the submission does not require an ethics statement.<br><br>General guidance is provided below. Consult the submission guidelines for detailed instructions. **Make sure that all information entered here is included in the Methods section of the manuscript.**	N/A

**Format for specific study types**<br><br>**Human Subject Research (involving human participants and/or tissue)**<br>• Give the name of the institutional review board or ethics committee that approved the study<br>• Include the approval number and/or a statement indicating approval of this research<br>• Indicate the form of consent obtained (written/oral) or the reason that consent was not obtained (e.g. the data were analyzed anonymously)<br><br>**Animal Research (involving vertebrate animals, embryos or tissues)**<br>• Provide the name of the Institutional Animal Care and Use Committee (IACUC) or other relevant ethics board that reviewed the study protocol, and indicate whether they approved this research or granted a formal waiver of ethical approval<br>• Include an approval number if one was obtained<br>• If the study involved *non-human primates*, add *additional details* about animal welfare and steps taken to ameliorate suffering<br>• If anesthesia, euthanasia, or any kind of animal sacrifice is part of the study, include briefly which substances and/or methods were applied<br><br>**Field Research**<br><br>Include the following details if this study involves the collection of plant, animal, or other materials from a natural setting:<br>• Field permit number<br>• Name of the institution or relevant body that granted permission	
**Data Availability**<br><br>Authors are required to make all data underlying the findings described fully available, without restriction, and from the time of publication. PLOS allows rare exceptions to address legal and ethical concerns. See the PLOS Data Policy and FAQ for detailed information.	Yes - all data are fully available without restriction

A Data Availability Statement describing where the data can be found is required at submission. Your answers to this question constitute the Data Availability Statement and **will be published in the article**, if accepted.<br><br>**Important:** Stating 'data available on request from the author' is not sufficient. If your data are only available upon request, select 'No' for the first question and explain your exceptional situation in the text box.<br><br>Do the authors confirm that all data underlying the findings described in their manuscript are fully available without restriction?	
**Describe where the data may be found in full sentences. If you are copying our sample text, replace any instances of XXX with the appropriate details.**<br><br>• If the data are **held or will be held in a public repository**, include URLs, accession numbers or DOIs. If this information will only be available after acceptance, indicate this by ticking the box below. For example: *All XXX files are available from the XXX database (accession number(s) XXX, XXX.*).<br>• If the data are all contained **within the manuscript and/or Supporting Information files**, enter the following: *All relevant data are within the manuscript and its Supporting Information files.*<br>• If neither of these applies but you are able to provide **details of access elsewhere**, with or without limitations, please do so. For example:<br><br>*Data cannot be shared publicly because of [XXX]. Data are available from the XXX Institutional Data Access / Ethics Committee (contact via XXX) for researchers who meet the criteria for access to confidential data.*<br><br>*The data underlying the results presented in the study are available*	All relevant data are within the manuscript and its Supporting Information files.

Powered by Editorial Manager® and ProduXion Manager® from Aries Systems Corporation

from (include the name of the third party and contact information or URL).<br>• This text is appropriate if the data are owned by a third party and authors do not have permission to share the data.<br><br>* typeset	
Additional data availability information:	

Manuscript
Click here to access/download;Manuscript;Manuscript.docx

Formulation of substrates with agricultural and forestry wastes for Camellia oleifera Abel seedling cultivation

Fei Zhou[1¶],Nianjin Wang[2¶],Jinping Zhang[1*],Xiaohua Yao[1],Tiantian Zhang[1],Xiaofeng Zhang[3]，Lingyan Zhan[4],Jieman Li[4]

[1]Research Institue of subtropical Forestry, Chinese Academy of Forestry, Hangzhou, Zhejiang, China

[2]Chun 'an County Forestry Bureau,Hangzhou, Zhejiang, China

[3]Fuyang District Environmental Protection Bureau, Hangzhou , Zhejiang, China

[4]Taihu County Huayuan Agricultural Science and Technology Development Co. LTD,Anqing,Anhui, China

* jinpingzhang@126.com

Abstracts： Five Camellia oleifera Abel seedling substrates were prepared using the conventional formula, but with the peat substituted by the composts of Camellia oleifera shell, pine chips, palm fiber residues, chicken manure, and sheep manure. The physical and chemical properties of the prepared substrates before and after seedling cultivation were determined and their effects on the growth of Camellia oleifera seedling were analyzed. It was found that the survival rates of the one-year-old seedlings produced from stem cuttings on all substrates at 6 months were greater than 97.5%. As compared with the conventional substrate, the substrates formulated with the composts were able to promote the seedling growth upon height, ground diameter, root length and root volume. The substrate prepared with the compost of Camellia oleifera shell+ palm fiber residue[illegible]hicken manure (A3), vermiculite and perlite (6:3:1) was the most optimal, which gave 100% seedling survival rate, the greatest seedling height, and the largest ground diameter. In particular, the ground diameters and 26.67% of the seedling heights reach the grade 1 standard for two-year-old seedlings.

Key words： Cultivation substrate; substrate; agricultural and forestry waste; Camellia oleifera; physical and chemical properties

1. Introduction

In China, 2 billion tons of a variety of agricultural and forestry wastes are produced every year. These wastes are renewable and biodegradable biomasses, yet only a small portion is reused. The majority of them are incinerated, buried, or disposed randomly, which is the waste of resources and causes environmental pollutions (Guo et al., 2021; Zhao et al., 2021; Lu et al., 2021). Agricultural and forestry wastes can be reutilized as the cultivation substrates for flowers, seedlings, and vegetables after treated by high-temperature aerobic fermentation (Zhao et al., 2021). Such applications not only re-

utilize biomass wastes and reduce environmental pollutions, but also effectively improve the ecological environment (Nie, 2017) and realize carbon sequestration.

Camellia oleifera Abel. is an important woody oil tree species in southern China (Zhuang, 2008). The rapid development of tea tree oil processing industry has dramatically increased the production of by-products, such as Camellia oleifera shells. Composting these by-products can not only prevent wasting resources and environmental pollution, but also provide substrates and organic fertilizers to improve soil quality and inhibit soil-borne diseases. Pot method with light substrates shows great advantages, such as all-season operation, high survival rates, no slow growth period, fast growth, less root damage during transplanting, long afforestation seasons, and low transportation costs for seedling production. (Wang et al., 2018; Liu et al., 2019; Lv et al., 2017; Yang et al., 2018). Substrate is one of the key factors affecting the performance of pot production of seedling (She et al., 2020). The conventional Camellia oleifera seedling substrates are formulated with topsoil and peat. Peat is a non-renewable resource, and its performance for Camellia oleifera seedling production can be further improved (Wu et al., 2017). Substituting peat with agricultural and forestry wastes has become a research hotspot. For example, Lu et al. reported the Camellia oleifera seedling production using the substrate formulated with the compost of forest wastes, weeds, and vermicompost (Lu et al., 2021). Wu et al. successfully obtained Camellia oleifera seedlings with the substrate containing the compost of sawdust, decomposed litter, biogas residue and yellow soil (Wu et al., 2017). Dai et al. also formulated the substrate with the compost of bagasse, cassava skin, peanut shells, charcoal ash, and garden soil to produce Camellia oleifera seedlings (Dai et al., 2016). All these reports used the composts of agricultural and forestry wastes as the substrates for the seedling production of Camellia oleifera, yet lacked the detailed information of the raw material sources, composting formula and composting method. The variation in the substrate raw material source makes it difficult to obtain consistent composts. The quality of Camellia oleifera shell compost has been successfully improved by adding different nitrogen sources, such as urea, compound fertilizer, and pig manure and EM bacteria (Luo, 2011), but no application research on the composts has been carried out.

Composting is a major process of recycling agricultural and forestry wastes. It is generally divided into aerobic composting and anaerobic composting. Aerobic composting decomposes organic matters under aerobic conditions mostly into CO_2 and water and releases heat. Anaerobic composting mainly produces methane, CO_2, and low molecular weight intermediates, such as organic acids, etc. Most conventional composting is conducted under anaerobic conditions, which tends to release odors (Li et al., 2017). The modern composting processes are mainly conducted under aerobic conditions with controlled water contents, C/N ratios and ventilation. Aerobic composting can transform unstable agricultural and forestry wastes into stable substances, during which pathogenic bacteria are killed. The composts thus can be safely handled and stored. They are also a good seedling substrate, soil conditioner and organic fertilizer (Zhang et al., 2021).

In this work, aerobic composting was conducted with Camellia oleifera shells, pine chips, palm fiber residues, chicken manure and sheep manure as the main raw materials using different formulas. The obtained composts were respectively mixed with boiler slag, vermiculite, and perlite at certain ratios as the substrates to grow Camellia oleifera seedlings. The changes in the physical and chemical properties of the substrates after seedling cultivation and the relationship between composting raw materials and the seedling development were comprehensively evaluated and analyzed, aimed to optimize a composting formula and substrate formula for the cultivation of Camellia oleifera seedling and provide experimental data and a scientific reference for the recycling and composting of agricultural and forestry wastes including Camellia oleifera shells as the substrate exclusively for the pot cultivation of Camellia oleifera seedlings.

2. Materials and methods

2.1 Compost materials and preparations

Camellia oleifera shells, palm fiber residues, chicken manure, pine chips, and sheep manure were provided by Anhui Wanxiuyuan Ecological Agriculture Group Co., Ltd., (Anqing, Anhui Province, China). The Camellia oleifera shells and pine chips were smashed to the sizes of 5-8 mm before use. The compost bacteria were purchased from Yijiayi Biological Engineering Co., Ltd. (Zhengzhou, China) and urea was obtained from Henan Jinkai Chemical Investment Holding Group Co., Ltd (Zhengzhou, China). The primary properties of the compost raw materials are listed in Table 1.

Table 1 Primary properties of compost raw materials

2.2 Composting process

Composting was conducted by window composting in the composting plant of Anhui Wanxiuyuan Ecological Agriculture Group Co., Ltd. from August to November 2020. Four composting piles including A1: Camellia oleifera shell + urea (C/N 60); A2: Camellia oleifera shell + sheep manure (C/N 55); A3: Camellia oleifera shell + palm fiber residue + chicken manure (C/N 55) /N 30); and A4: pine chips + chicken manure (C/N 40.7) were prepared, mixed, and stirred evenly. The moisture content of each pile was adjusted to 55-60% and 0.1% fermentation bacteria were added into each pile. Each pile was then thoroughly mixed, stirred evenly, and stacked for high-temperature aerobic fermentation at the same time. The composting piles were turned for aeration and sampled by the five-point sampling method every 7 days. For each sample, half portion was stored at -20℃ and the other half was dried at 65℃ and ground.

2.3 Compound substrate preparation and seedling cultivation

Seedlings were cultivated at the nursery of Anhui Wanxiuyuan Ecological Agriculture Group Co., Ltd. located in Taihu County, Anqing, Anhui Province between 30°09' N to 30°46' N and 115°45 'E to 116°30 'E. The temperatures during the experiment varied between from -6 °C and 34 °C, and there were 24 days with temperatures lower than 0 °C.

Compound substrates were prepared with the composts obtained above and inorganic matrices using the formulas shown in Table 2. In December 2020, one year old Camellia oleifera seedlings with intact root systems, the heights of 13 ± 3 cm and ground diameters of 0.22 ± 0.02 cm were respectively transplanted into pots with the diameter of 16 cm and height of 16 cm. The pots were filled with the compound substrates listed in Table 2. For each substrate, 200 seedlings were planted and allowed to grow for 6 months. The substrates were then sampled and analyzed for physical and chemical properties. During the seedling cultivation, the substrates were maintained moist, and watered thoroughly when they became dry during the fast growing period.

Table 2 Formulas of compound substrates

2.4 Analytical methods

During composting, the compost temperature was measured from the upper (10 cm from the top), middle and lower (10 cm from the bottom) parts of the pile at around 3:00 pm every day, and the ambient temperature of the day was recorded at the same time. The bulk density, total porosity, aeration porosity, water-holding porosity, electrical conductivity (EC) and pH were also measured following the Chinese Forestry Industry Standard GB/T 33891-2017 of organic substrates for greening. Total organic carbon (TOC), total nitrogen (TN), total phosphorus (TP), total potassium (TK) and germination index (GI) were determined by the method of Zhang et al. (2018). The organic matter content, total nutrient content, C/N ratio and survival rate of Camellia oleifera seedling were calculated with Eq. (1), (2), (3) and (4), respectively.

$$\text{Organic matter (\%)} = \text{TOC (\%)} \times 1.724 \quad (1)$$

$$\text{Total nutrient (\%)} = \text{TN (\%)} + P_2O_5\ (\%) + K_2O\ (\%) \quad (2)$$

$$C/N = TOC(\%)/TN(\%) \quad (3)$$

$$\text{Survival rate} = \text{number of survived seedlings} \div \text{total number of planted seedlings} \times 100\% \quad (4)$$

After 185 days of cultivation, 60 seedlings were randomly collected from each group and measured for ground diameter and height. Five seedlings were randomly selected from each group and analyzed for root length, surface area, volume and average diameter using a Wanshen LA-S root system analyzer to evaluate the effects of the physical and chemical properties of substrate on the growth of Camellia oleifera

seedling. The obtained data were processed and analyzed using the EXCEL and SPSS softwares.

3. Results and discussion

3.1 Compost temperature

Temperature is one of the important indicators to evaluate the compost maturity (Gu et al., 2015; Bustamante et al., 2008). A composting process is generally divided into four phases, e.g., the mesophilic phase, the thermophilic phase (>50 °C), the cooling phase, and the maturation phase. Pathogenic microorganisms, insect eggs and weed seeds are killed at the thermophilic phase when the compost temperature increases to over 55 ℃, and thus compost becomes harmless. The fermentation bacteria are rapidly deactivated as temperature increased to over 63 ℃, and the compost tempeture drops, leading to cooling phase (Bernal et al. 2009). As can be seen from Figure 1, the temperatures of all compost piles are much higher than the ambient temperature during the composting, indicating that ambient temperature has little effects on the composting. The initial temperatures of all composting piles were 27±0.6 ℃. The temperatures of A1, A2, A3 and A4 increased to above 50℃ on the day 6, 7, 3 and 3, respectively, and remained at 50 ℃ for 20, 23, 39, and 9 days, and at 60 °C for 4, 7, 22, and 6 days, respectively, conforming to the safety and hygiene requirements (Wang et al., 2017). The four compost piles entered the cooling phase on day 33, 34, 44 and 15, respectively and their maturation phases lasted 38, 42, 44 and 21 days, respectively. The sudden temperature drops are due to heat dissipation during manual turning for aeration (Qian et al, 2014; Guardia et al, 2010a).

Figure 1 Temperature changes of the four compost piles during composting

The compost pile A3 exhibited the largest heating rate, the highest high temperature, and the longest thermophilic phase. It can be explained that, first, the initial C/N (30) of A3 is most suitable for microbial reproduction (Bernal et al. 2009). Second, there is a large amount of chicken manure in the pile. Chicken manure is rich in protein and urea that can be easily decomposed and utilized by microorganisms. Third, the palm fiber residues can improve the aeration porosity of the compost, which is conducive to the proliferation of the microorganisms and heat generation. Piles A1, A2 and A3 all contain Camellia oleifera shells, but have different C/N ratios due to different excipients. The C/N ratio of A3 is 30, which is in the optimal range (25-35), while those of A1 and A2 are 60 and 55, respectively, that indicate low nitrogen contents. The activity of microorganisms is lower under the insufficient nitrogen conditions, and thus the composting reactions are slow (Ma et al., 2020). The temperature of pile A4 increased rapidly and thus its initial temperature was higher. However, it entered the cooling phase earliest and its temperature dropped fastest. It can be explained that the nutrients in the chicken manure are rapidly decomposed. The main components in pine chip are cellulose and lignin that are difficult to be degraded and the hemicellulose content is only 27.59%. In contrast, the Camellia oleifera shells in other three groups are rich in highly degradable hemicellulose (49.34%), which can prolong the thermophilic phase. These results suggest that the physical and chemical

properties of raw materials directly affect the composting process and the physical and chemical properties of compost (Table 3).

3.2 *Physical and chemical properties of composts and substrates*

The composts of agricultural and forestry wastes can be used alone as a seedling substrate or can be formulated with other matrices to form different seedling substrates. The substrates with large bulk densities are favorable for the seedling handling. The cohesions of the substrates with small bulk densities are poor, which is inconducive to the root fixation (Li et al., 2020). The bulk densities of A1, A2, A3, and A4 composts are 0.39 g·cm^{-3}, 0.40 g·cm^{-3}, 0.47g·cm^{-3}, and 0.42 g·cm^{-3}, all within the ideal bulk density range of 0.1-0.8 g·cm^{-3} (Wang et al., 2021). Porosity is another important physical property that affects the aeration, drainage and water holding capacity of a substrate. The total porosities of A1, A2 and A3 composts are 73.94%, 74.13% and 65.90%, respectively, which are all within the ideal total porosity range of seedling substrate (40%-75%) (Wu, 2006; Wang et al., 2013). The total porosity of A4 compost (83.4%) is too high as a seedling substrate and can be adjusted with other matrices. The pHs, seed germination indexes (GIs), and organic matter contents of the composts are in the ranges of 7-8, 89.12-99.42, and 65.86-81.55%, respectively, which meet the standard NY/T525-2021 for organic fertilizer defined by the Ministry of Agriculture and Rural Affairs of the People's Republic of China (pH 5.5-8.5, GI≥70%, and organic matter content≥30%). Germination index (GI) is a parameter that can be used to quickly evaluate the phytotoxicity of a compost in a short period of time and is of great importance in actual productions. The GIs of all composts are >85%, and thus the composts can be considered non-toxic to plants (Yamamoto, N. et al. 2011). The total nutrient contents of A1, A2, A3, and A4 composts are measured to be 3.27%, 3.85%, 6.32%, and 1.80%, respectively. That of A3 compost reaches the standard NY/T525-2021 for organic fertilizer (≥4%) and thus it can be used as an organic fertilizer or a substrate component. The rest 3 groups can be used as cultivation substrates or substrate raw materials.

Table 3 Physical and chemical properties of the composts of agricultural and forest waste and other components for the preparation of compound substrates

3.3 Changes in physical and chemical properties of compound substrates after seedling cultivation

The water holding capacities of the obtained composts are low, and thus vermiculite with a better water holding ability is introduced. The raw materials listed in Table 3 were mixed at the ratios listed in Table 2, and the physical and chemical properties of the corresponding compound substrates were measured (Table 4). The bulk densities of compound substrates A, B, C, D, and E were measured to be 0.41 g·cm^{-3}, 0.47 g·cm^{-3}, 0.41 g·cm^{-3}, 0.42 g·cm^{-3}, and 0.41 g·cm^{-3}, respectively and their total porosities were 73.83%, 67.83%, 66.70%, 68.17% and 74.60%, respectively, all within the ideal ranges of 0.1-0.8 g·cm^{-3} and of 40%-75% (Wang et al., 2021 ; Wu,

2006; Wang et al., 2013). Their aeration porosities (29.31%, 28.12%, 25.44%, 24.22% and 23.10%), pH (7.03-7.43) and EC values (0.12-0.20 ms·cm^{-1}) all conform to the national standard "Organic Substrates for Greening" (GB/T 33891-2017) of the People's Republic of China with the requirements of total porosity≥20%, pH 5.0-7.6 and EC≤0.65 ms·cm^{-1}. Yet none of the pHs conforms to the Forestry Industry Standard LYT 2314-2014 "Technical Regulations for Seed Box Production of Camellia Container Seedling" of the People's Republic of China (pH 5.0-6.5).

The changes in the physical and chemical properties of the five compound substrates after the seedling cultivation are summarized in Table 4 and 5. As can be seen, the bulk densities, aeration porosities and EC values of all groups increase, while their total porosities and water-holding porosities, organic matter contents and total nutrient contents decrease after the 6-month cultivation. It can be explained that, first, most of the hemicellulose in the compost raw material is degraded during composting (Zhang et al., 2019), and large amounts of refractory cellulose and lignin continue to decompose slowly during the cultivation, which increases the aeration porosity. Second, with the seedling growth and development, the root elongation squeezes the substrate and thus decreases the total porosity and water-holding porosity. Third, the seedling growth consumes large amounts of organic matters, nutrients, and minerals, which decreases the contents of organic matters, nitrogen, P_2O_5, K_2O and total nutrient. The seedling development also accelerates the decomposition of refractory substances, resulting in increased EC values. The seedlings of group C show the highest survival rate, the greatest seedling height, and the largest ground diameter (Table 6), and the aeration porosity of the substrate is increased by up to 104.05%. The total porosities of substrates A, B, C, D and E decrease by 21.35%, 13.31%, 7.95%, 5.38% and 20.11%, respectively, after the cultivation. Both substrates C and D contain the palm fiber residue a high lignin content (Table 1). Lignin is difficult to be degraded, which explains the smaller changes in the total porosity of two substrates. Therefore, it can be concluded that the substrate porosity changes after seedling cultivation are related to the cellulose and lignin contents of the compost feedstock. Substrates A, B, C and D all contain the Camellia oleifera shell with a high hemicellulose content (Table 1). The compost in substrate A was abstained in the absence of livestock or poultry manure and the microbial abundance (Jinping et al. 2021) and temperature of the compost were lower than those of the compost in substrate B (Figure 1). Therefore, the hemicellulose degradation rate of substrate A is lower than that of substrate B (Zhang et al. 2021). The hemicellulose in substrate A was further degraded during the seedling cultivation, leading to the largest decrease in total porosity. The cellulose content of pine chips in substrate E is high (Table 1). Cellulose is difficult to be decomposed, but easier than lignin. Therefore, small amounts of hemicellulose, cellulose and lignin continue to decompose during the seedling cultivation, which causes the significant decreases in the total porosity of substrate E.

Table 4 Changes in the physical and chemical properties of compound substrates after seedling cultivation

Table 5 Changes in the nutrient contents of compound substrates after seedling cultivation

3.4 *Relationship between physical and chemical properties of compound substrate and seedling morphology*

Survival rate is an important indicator for evaluating the successful cultivation of seedlings. The seedling survival rates of all groups are above 97% (Table 6), significantly higher than that (42.50-67.50%) obtained using the compost of rice hulls, bark, and sawdust (rice hulls 20%-26%, bark 28%-44%, sawdust 36%-52%) as the substrate (Lu et al., 2017).

Seedling height and ground diameter are important indicators of seedling quality (Wang et al., 2021). As shown in Table 6, the average heights and maximum heights of the seedlings cultivated on the 5 substrates for 6 months are in the order of C> D>B>E>A and C>B>D>A>E, respectively. Both of their average ground diameters and maximum ground diameters are in the order of C>D>B>E>A. The average ground diameter (4.1 mm) and average seedling height (31.4 cm) of group C respectively reach the first-grade and second-grade criteria defined in the standard GB/T 26907-2011 for two years old seedling (≥0.35 cm and ≥25 cm). In addition, 26.67% of the seedling heights reach the first-grade criteria (≥40 cm), and 45% of them reach the second-grade criteria. The average ground diameters of both groups B (3.7 mm) and D (3.9 mm) reach the first-grade criteria. Upon their seedling heights, 6.7% of group B and 15% of group D reach the first-grade criteria and the rest all reach the second-grade criteria. The average ground diameter (3.3 mm) of the seedlings in group E meets the second-grade criteria (≥0.30 cm), but only 28.3% of the seedling heights meet the second-grade criteria. Neither of the average ground diameter (2.8 mm) nor the average seedling height (20.0 cm) of the seedlings in group A conforms to the second-grade criteria, but 41.67% of the seedlings meet the second-grade standard for ground diameter and 15 % of them meet the second-grade standard for height. It is worth noting that the average seedling height of the seedlings in group C (31.4 cm) is 30.5 cm higher than that of two-year-old Camellia oleifera seedlings cultivated on the substrate prepared with coconut bran and yellow soil (coconut bran 75% +yellow soil 25%) and 3 kg/m^3 of slow-release compound fertilizer from KOCH (USA) (Wang et al., 2018). The average seedling heights (31.4 cm, 29.6 cm) and average ground diameters (4.1 mm, 3.9 mm) of the seedlings in groups C and D are better than those (28.70 cm, 3.93 mm) of the seedlings raised on the substrate formulated with peat and the compost of rice hulls (peat 50% + (compost of rice hulls+ extruded perlite + vermiculite + boiler slag + imported slow-release fertilizer) 50%) for 18 months (Cui et al., 2017). The average seedling heights (27.2 cm, 31.4 cm and 29.6 cm) and average ground diameters (3.7 mm, 4.1 mm and 3.9 mm) of the seedlings in groups B, C and D are better than those (25.98 cm, 3.43 cm) of the seedlings raised on the substrate containing composted bagasse, cassava skin and charcoal ash (2:1:1) for 18 months (Dai et al., 2016).

The compound substrate C contains the highest amounts of N, P_2O_5, K_2O, and total nutrients before seedling cultivation, and the decreases in the contents of N, K_2O, and

total nutrient and the increase in aeration porosity are the most significant after the seedling cultivation (Table 5 and 6). The seedling survival rate, height, and ground diameter of this group are also the greatest. These results suggest that seedling height and ground diameter are mainly related to the total nutrient content of the substrate, consistent with the results reported by Wu et al. and Zeng et al. (Zeng et al., 2021; Wu et al., 2018). Compared with those of other substrates, the N and K_2O contents of substrate C decrease most significantly, by 70.31% and 70.42% respectively, while the decrease in its P_2O_5 content is the smallest, suggesting that nitrogen and potassium can promote the growth and development of Camellia oleifera seedling (Chen, 2009; Wang et al., 2010; Peng, 2019).

The organic matter contents of substrates A-E decreased by 82.61%, 62.40%, 66.44%, 73.16% and 74.37%, respectively, after the seedling cultivation due to the consumption by the seedlings and losses during watering. Before the seedling cultivation, the organic matter contents and total nutrient contents of the substrates are in the order of E>A>B>C>D, and C>D>B>A>E, respectively. The substrates with high organic matter contents generally show low total nutrient contents. The raw materials of the composts in substrates C and D are the same, but their formulas and ratios with other inorganic matrices are different. The ratio of the compost in substrate C is 20% higher than that in substrate D. Therefore, both the contents of organic matter and total nutrient of substrate C are higher than those of substrate D. The seedling heights and ground diameters of groups B, C, and D are significantly greater than those of the other two groups, but the decreases in the organic matter contents of their substrates are smaller, indicating that there are large amounts organic matters in the composts of Camellia oleifera shell and livestock/poultry manures that can be absorbed and utilized by the seedlings. The maximum decrease in the total nutrient content of substrate C is only 3.43%, and the average seedling height and ground diameter of the seedlings raised on it are the largest. Even though the organic matter content of substrate A decreased by 82.61% after the seedling cultivation, it showed no significant advantages for the seedling growth. It may be explained with the low degradation rate of lignocellulose during the composting that results in low contents of the organic matters usable for the seedling development. All these results suggest that the height and ground diameter of Camellia oleifera seedling are closely related to the properties of compost raw materials, composting formula, composting process and rate, and substrate formula. The stable compost raw materials, formula, composting process, and substrate formula are the keys to improving the quality and efficiency of the Camellia oleifera seedling cultivation.

Table 6 Morphologies of seedlings raised on different substrates

The physical and chemical properties of substrate also affect the morphological characteristics of Camellia oleifera seedling root system. Root morphology can be characterized with root growth indicators, such as root length, root surface area, root volume, and root diameter (Pang et al., 2018; Li et al., 2020). In general, the longer and thicker root systems with larger surface areas and volumes suggest better development

and growth conditions. As shown in Table 6, the root morphologies of the seedlings raised on different substrates are significantly different. The seedlings of group B exhibit the longest root system of 4377.77 cm, the largest root surface area of 768.81 cm^2, and the largest root volume of 10.86 cm^3, and both the root surface area and volume of group A are the smallest. The root surface areas and root volumes of the seedlings raised on the five substrates are in the orders of B>E>D>C>A and B>D>E>C>A, respectively. The average root diameter of group D is 0.66 cm, significantly larger than those of other groups (0.56 cm for groups A, B and E, and 0.58 cm for group C). Despite the larger root length, surface area and volume of group B, its average root diameter is similar to those of other groups. Yet the overall root development of group B is the best, possibly because the porosity and nutrient content of its substrate is more suitable for the root development of Camellia oleifera seedling. The total root lengths, surface areas, and volumes of all five groups are better than those of the Camellia oleifera seedlings raised on conventional substrates. For example, the seedlings grown on the substrate formulated with 40% yellow soil + 15% pine forest topsoil + 20% mushroom residue + 20% peat + 5% manure for two years only show the average total root length of 280.32 cm, the surface area of 106.72 cm^2, and the average root volume of 8.71 cm^3 (She et al., 2020).

3.5 *Correlation analysis of seedling morphology and substrate physical and chemical properties*

Table 7 Pearson correlation analysis of physical and chemical properties of substrate and seedling morphology

Seedling height, ground diameter and root activity are generally considered to be the most important indicators of seedling quality (Lu et al., 2002; Ge et al., 2006). Herein, we evaluated the Camellia oleifera seedling growth with seedling height, ground diameter and root morphology and analyzed the correlation between the physical and chemical properties of substrate and the seedling growth. As can be seen from Table 7, the moisture content of substrate is negatively correlated to seeding ground diameter (r=-0.972, P<0.01) and seedling height (r=-0.997, P<0.01). Seedling height and ground diameter are positively correlated (r=0.982, P<0.01), and root surface area and root length are positively correlated (r=0.971, P<0.01). The total porosity of substrate before seedling cultivation is negatively correlated to seedling height (r=-0.891, P<0.05) and ground diameter (r=-0.944, P<0.05). The changes in the contents of organic matter and total nutrient of substrate after seedling cultivation reflect the amounts of substrate nutrients absorbed by the seedlings and the losses of the substrate itself. The change in organic matter content is negatively correlated to ground diameter (r=-0.905, P<0.05) and seedling height (r=-0.957, P<0.05), and is positively correlated to moisture content (r=0.966, P<0.01), aeration porosity seedling cultivation (r=0.952, P<0.05) and organic matter content before seedling cultivation (r=0.906, P<0.05). The change in total nutrient content is positively correlated to the total nutrient content before seedling cultivation (r=0.983, P<0.01). The total nutrient content after seedling cultivation is negatively correlated to the aeration porosity before

seedling cultivation (r=0.937, P<0.05) and is positively correlated to the total nutrient content before seedling cultivation (r=0.935, P<0.05). No correlation is found between the change in total nutrient content and seedling ground diameter (r=0.732) and seedling height (r=0.773). There are no significant correlations between the contents of organic matter and total nutrient before and after seedling cultivation and seedling ground diameter and height (r1=-0.723, r2=-0.823, r3=0.304, r4=184; r5=0.754, r6=0.820, r7=0.723, and r8=0.830). These results suggest that organic matters and nutrients that can be absorbed and utilized by Camellia oleifera seedling play a key role in the seedling growth and development.

4 Conclusion

Agricultural and forestry residues were composted using different formulas and the composting efficiencies were evaluated. It is found that the introduction of livestock and poultry manures and the C/N ratios in the range of 25-35 can significantly increase the heating rate, prolong the thermophilic phase, and produce the composts with high total nutrient contents and GIs.

Five compound substrates were prepared using the formulas containing the obtained composts and inorganic substrates, vermiculite and perlite, with no peat added for the cultivation of one year old Camellia oleifera seedlings. The physical and chemical properties of the substrates before and after seedling cultivation and the seedling development were analyzed. The results show that, first, the physical and chemical properties of compost raw materials, composting formula and degree of composting directly affect the physical and chemical properties of the obtained compost and corresponding compound substrate, as well as the seedling growth. Second, the physical and chemical properties of compound substrate conform to the standards defined in GB/T 33891-2017 for seedling substrate, except that their pHs (7.03-7.43) do not meet the pH requirement (5.0-6.5) of the regulation LYT 2314-2014. However, the Camellia oleifera seedlings developed better on these substrates than on the traditional nursery substrates. Therefore, further study is needed to evaluate the effects of substrate pH on the growth of Camellia oleifera seedling. Third, the survival rates of the seedlings cultivated on the five substrates for 185 day covering the severely hot summer and old winter are all greater than 97.5%. In particular, the seedlings cultivated on substrate C with the highest total nutrient content exhibit 100% survival rate, and the largest seedling height and ground diameter. In addition, all of the seedling heights and 26.67% of ground diameters reach the first grade criteria for two years old seedling. Forth, the changes in the physical and chemical properties of substrate after seedling cultivation are correlated to the growth and development of Camellia oleifera seedling. The initial total porosity is negatively correlated to seedling height at the significant level, and the change in organic matter content is negatively correlated to ground diameter and seedling height, and positively correlated to the initial aeration porosity and initial organic matter content at significant levels. Based on these results, it can be concluded that the amounts of organic matters and nutrients in the substrate that can be absorbed and utilized by seedling play a key role in the seedling development.

Acknowledgments

The authors would like to thank Changsheng Zhan (Taihu County Huayuan Agricultural Science and Technology Development Co. LTD,Anqing,Anhui, China) for the nursery management and site support.

References

[1] Dai X, Sun W, Fan Q, Luo P, He J. Physicochemical property of mixed substrates with agricultural and forestry wastes and comprehensive evaluation of their effect on growth of Camellia oleifera seedlings.Journal of Plant Resources and Environment. 2016; 25(1):54-61.doi: 10.3969/j.issn.1674-7895.2016.01.07

[2] Guo W, Qi W, Wang Z, Wang C. Analysis on the research status and development of agriculture and forestry waste resource utilization under the condition of blending and briquetting. Journal of China Agricultural University. 2021; 26(1):143-150.doi: 10.11841/j.issn.1007-4333.2021.01.15

[3] Zhao Y, Liu J, Zhang B. Effects of Different Substrate Ratio of Agricultural and Forestry Waste and Slow-release Fertilizer on the Growth of Ornamental Pepper. Horticulture & Seed. 2021; 41(09):6-9. doi: 10.16530/j.cnki.cn21-1574/s.2021.09.003

[4] Lu H, Chen W, Su Y, Xie P. Research on fertilizer effect of forest waste compost as seedling substrate. Rural Economy and Technology. 2021;32(10):17-20.

[5] Nie L. Research on the present situation and development strategy of Agricultural waste resource recovery in China. Agricultural Economic Research. 2017; 07:59-60.

[6] Wu Q, Chen J, Gan F, Li C, Jiang H, Zheng H.Application of Well-composted Sawdust and Litterfall Substrate to Camellia oleifera Seedling. Guangxi Forestry Science. 2017; 46(4):431-435. doi: 10.19692/j.cnki.gfs.2017.04.021

[7] Luo J, Tan X, Peng Y. A Study on the Composting Technology for Camellia Shell Medium. Acta Agriculturae Universitatis Jiangxiensis. 2011; 33(4):0712-0718.doi: 10.13836/j.jjau.2011127

[8] Zhuang R. China camellia.Beijing: China Forestry Publishing House, 2008.

[9] Wang M, Zhang Y, Tang X. Report of Light Media Nursery Test For Camellia. Forestry and Environmental Science. 2018; 34(6):56-60.

[10] Zhang X, Ding X, Zhang Y, Liu Y, Cai J, Li Y. Effects of nutrient defifi ciencies on root system in Camellia gauchowensis seedlings. Nonwood Forest Research. 2014; 32(4):170-174. doi: 10.14067/j.cnki.1003-8981.2014.04.033

[11] Wang Q, Peng G. Influence of substrate and time on cuttage steckling of Camellia oleifera. Forest By-Product and Speciallity in China. 2021; 04:001-003.doi: 10.13268/j.cnki.fbsic.2021.04.001

[12] Li Y, Ma J, Wang D, Wei Y, Ye H. Effects of Mediums with Different Physicochemical Properties on Cutting Rooting of Camellia osmantha.Chinese Journal of Tropical Agriculture. 2020;40(3):25-30. doi: 10.12008/j.issn.1009-2196.2020.03.005

[13] Lv B, Jiang M, Ye F, Zheng X, Yu S. Research on matrix formula of oil tea container seedling. South China Agriculture. 2017;11(5):24-26. doi:

10.19415/j.cnki.1673-890x.2017.05.014

[14] Yang L, Chen W, Chen X, Chen J, Weng C, Zheng D. Effects of different light substrates and containers on rooting of camellia oil cuttings and evaluation of matrix bagging methods. Modern gardening. 2018;10:43-45. doi: 10.14051/j.cnki.xdyy.2018.19.022

[15] Liu C, Li J, Shu Q. Growth Comparison of Camellia oleifera Seedlings Indoor and Outdoor with Different Substrates. Guangxi Forestry Science. 2019; 48(3):332-335. doi: 10.19692/j.cnki.gfs.2019.03.010

[16] She Y, Zhang Y, Cao K, Li X, Zhang L, Wang Y. Effects of different culture medium formulas on roots biomass and morphology of Camellia oleifera container seedling. Non-wood Forest Research. 2020;38(4):11-16. doi: 10.14067/j.cnki.1003-8981.2020.04.002

[17] Hua M, Wang J. Experimental instruction of soil physics. Beijing: Beijing Agricultural University Press. 1993;61-73.

[18] GB/T 33891-2017, Organic substrates for greening. Beijing: Standards Press of China, 2017.(in Chinese)

[19] Shuang L, Ni S, Du J, Teng Z, Shi C. Determination of Organic Carbon in Geochemical Soil Sample by Potassium Dichromate Oxidation-Heating Method. Anhui Chemical Industry. 2016; 42(4): 110-112.

[20] Bremner JM Nitrogen-total. In: Sparks, D.L. (Ed.), Methods of Soil Analysis. Part 3 – Chemical Methods. SSSA Inc., ASA Inc., Madison, WI, USA, pp. 1996;1085-1122.

[21] Tiquia SM, Tam NFY and Hodgkiss IJ Effects of turning frequency on composting of spent pig-manure sawdust litter. Bioresource Technology 1997; 62(1-2): 37-42.

[22] Wang Y, Li M, Qiu H, Zhang W, Zhang C, Li Y. Changes of microbial quantity and nutrient content in different composting of livestock manure. Journal of Gansu Agricultural University. 2017;52(3), 37-45. doi: 10.13432/j.cnki.jgsau.2017.03.007

[23] Qiao X, Shen G, Wang Z, Guo C, Liu Y, Lei Z, et al. Co-composting of livestock manure with rice straw: Characterization and establishment of maturity evaluation system. Waste management, 2014;34(2):530-535.doi: 10.1016/j.wasman.2013.10.007

[24] Guardia A.D, Mallard P, Teglia C, Marin A, Pape C.L, Launay M, et al. Comparison of five organic wastes regarding their behaviour during composting: part 1, biodegradability, stabilization kinetics and temperature rise. Waste Management. 2010; 30(3):402-414. doi: 10.1016/j.wasman.2009.10.019

[25] Ma R, Li D, Qi C, Li G, Wang G, Liu Y, et al. Effects of C/N ratio on compost maturity and odor emission of chicken manure. Transactions of the Chinese Society of Agricultural Engineering. 2020; 36(24):194-202. doi: 10.11975/j.issn.1002-6819.2020.24.023

[26] Wang Q, Liu G, Zhang R, Li Y, Liu A, Wang W. Effects of Physical and Chemical Properties of the Substrate on the Growth of Container Seedlings of Ficus religiosa. Journal of Northwest Forestry University. 2021;36(5):88-93. doi: 10.3969/j.issn.1001-7461.2021.05.1

[27] Wu J. Comparison of physical and chemical properties of several solid cultivation substrates. Journal ofJilin Agricultural Sciences. 2006;31(4):17- 20.doi: 10.16423/j.cnki.1003-8701.2006.04.006

[28]LYT 2314-2014.Technical specification for seed raising of oil tea containers.Beijing: State Forestry Administration, 2014. (in Chinese)

[29]Wu J, Li X, An S. Effects of Different Substrate Ratios on the Growth of Sour Pummelo Seedlings. Chinese Journal of Tropical Crops. 2018;39(3):443-447. doi: 10.3969/j.issn.1000-2561.2018.03.006

[30]Zeng L, Li J, Wu F, Huang S, Li M, Cai Y. Effects of different substrate formulations on substrate nutrient content and seedling growth of Macadamia nut. Northern Horticultur. 2021; 24:51-56. doi: 10.11937/bfyy.2021135

[31]Chen H. Studies on factors concerned in nutrient media aeration improving growth as well as yield in cucumber. Shenyang,Liaoning, Shenyang Agricultural University, 2009.

[32]Wang Y, Shao X, Huang X, Wang K. Advances in studies on nitrogen uptake in plant roots. Pratacultural Science. 2010;27(7):105-111.

[33]Peng Z. Absorption, transportation and regulation of nitrogen element in plants. Journal of Hebei Agricultural University. 2019; 42(2):1-5. doi: 10.13320/j.cnki.jauh.2019.0024

[34]Ma S. Effects of Application Amount and Fertilization Modes of N,P and K on Growth,Yield and Quality of Eggplants. Shandong, Shandong agricultural University, 2018.

[35]Li C. Effects of Nitrogen,Phosphorus and Potassium Fertilization on the Growth and Physiological Characteristics of Saraca Dives Seedlings.Guangxi, Guangxi University, 2020.

[36]Yin J. Effects of Nitrogen Rate and Ratio of Nitrogen,Phosphorus,and Potassium on Dry Matter Accumulation and Grain Filling of Castor Bean (Ricinus Communis L.). Jiangsu,Yangzhou University, 2019.

[37]Lu M, Jiang F, Song X. Assessing indices of container seedling quality. Chinese Journal of Applied Ecology. 2002;13(6):763-765. doi: 10.13287/j.1001-9332.2002.0181

[38]Ge Y, Zhu J, Jiang B, Yuan W, Shen A. Study on evaluation index of container seedling quality. Journal of Zhejiang Forestry Science and Technology. 2006; 26(1):10-12，22.

[39]Wang X, Lin X, Li Y, Ruan Y, Xing X. Physical and chemical properties of several kinds of agriculture and forestry waste composite matrix and their effect on container seedling of Phoebe chekiangensis. Journal of Zhejiang A & F University. 2013;30(5):674 - 680.

[40]Li J, Zhao X, Wei S, Zheng J, Hu Y. Study on the Physico-Chemical Properties of soil-Less Cultural Substrates of Pollution-Free Vegetable. Journal of Southwest Agricultural University. 2000;22(2):112-115. doi: 10.13718/j.cnki.xdzk.2000.02.006

[41]Pang S, Ma Y, Zhang P, Lao Q, Yang B, Liu S. Effects of Substrate Ratio and Applying Dosages of Slow-release Fertilizer on the Growth of Container Parashrea chinensis Seedlings. Journal of Northwest Forestry University. 2018;33(6): 66-71. doi: 10.3969/j.issn.1001-7461.2018.06.11

[42]Peng S, Chen Y, Yang X, Wang X, Ma L, Wang R, et al. Study on the Classification of Seedling Quality of Oil-tea Camellia (Camellia oleifera). Chinese Agricultural

Science Bulletin. 2012; 28(25): 101-105.

Tables and Figures

Table 1 Primary properties of compost raw materials

Raw material/Property	TOC (%)	TN (%)	C/N	Cellulose/%	Hemicellulose/%	Lignin/%
Camellia oleifera shells	51.80±2.58	0.729±0.03	71.359±3.74	18.62±0.22	49.34±0.07	29.71±0.14
Brown silk chips	46.80±2.23	2.68±0.14	17.557±1.76	28.15±0.18	20.58±0.08	44.08±0.16
Pine chips	54.20±2.74	0.285±0.01	190.964±5.19	48.36±0.11	27.59±0.12	19.58±0.21
Chicken mature	34.39±1.75	2.73±0.14	12.667±1.31	-	-	-
Sheep mature	36.47±1.81	2.78±0.14	13.186±1.32	-	-	-
Urea	20±0.18	46.4±1.22	43.1±1.12	-	-	-

"-"indicates extremely low content or none

Table 2 Formulas of compound substrates

Treatments	Substrate formula
A	(Camellia oleifera shell + urea):Vermiculite: Perlite=6:3:1
B	(Camellia oleifera shell + sheep manure):Vermiculite: Perlite=6:3:1
C	(Camellia oleifera shell + palm fiber residue+chicken manure):Vermiculite: Perlite=6:3:1
D	(Camellia oleifera shell + palm fiber residue+chicken manure):Boiler slag:Vermiculite: Perlite=4:2:3:1
E	(Pine chip + urea):Vermiculite: Perlite=6:3:1

Table 3 Physical and chemical properties of the composts of agricultural and forest waste and other components for the preparation of compound substrates

Group	Moisture content MC/%	bulk density g·cm^{-3}	Total porosity %	Aeration porosity %	Water-holding porosity %	pH	EC (ms·cm^{-1})
A1	10.27±0.52	0.39±0.018	73.94±3.68	6.07±0.29	67.87±3.39	7.00±0.21	0.13±0.02
A2	9.46±0.48	0.40±0.021	74.13±3.70	9.30±0.47	64.83±3.23	8.00±0.26	0.08±0.03
A3	13.82±0.68	0.47±0.024	65.90±3.31	11.83±0.60	54.07±2.71	7.79±0.24	0.20±0.01
A4	22.47±1.11	0.42±0.02	83.40±4.16	1.80±0.089	81.60±4.07	7.00±0.22	0.06±0.03

Group	TN%	TK%	TP%	TOC%	total nutrient %	GI%	organic matter %
A1	1.07±0.05	1.83±0.09	0.37±0.02	38.20±1.92	3.27±0.15	89.12±4.44	65.86±3.25
A2	1.16±0.057	2.07±0.12	0.62±0.03	36.30±1.82	3.85±0.21	90.13±4.49	62.58±3.11
A3	2.75±0.14	2.55±0.11	1.02±0.05	29.80±1.48	6.32±0.33	99.42±4.98	51.38±2.54
A4	0.84±0.04	0.54±0.03	0.42±0.02	47.30±2.37	1.80±0.08	91.31±4.52	81.55±4.06

Raw materials	Moisture content MC/%	bulk density g·cm^{-3}	Total porosity %	Aeration porosity %	Water-holding porosity %	pH	EC(ms·cm^{-1})
Boiler slag	2.08±0.11	0.70±0.03	63.10±3.16	0.40±0.019	62.7±3.14	8.00±0.025	0.22±0.012
Vermiculite	1.55±0.078	0.13±0.01	95.04±4.75	30.03±1.51	65.01±3.24	7.00±0.021	0.047±0.0024
perlite	0.14±0.007	0.16±0.01	60.33±3.03	29.51±1.48	30.82±1.55	7.00±0.019	0.022±0.0011

Table 4 Changes in the physical and chemical properties of compound substrates after seedling cultivation

group		A	B	C	D	E
bulk density g/cm^3	before	0.41±0.02	0.47±0.02	0.41±0.02	0.42±0.02	0.41±0.02
	after	0.57±0.027	0.76±0.037	0.59±0.028	0.59±0.029	0.60±0.031
	increase	39.02±4.39	61.70±2.77	43.90±2.17	40.48±2.02	46.34±2.20
total porosity %	before	73.83±3.65	67.83±3.34	66.70±3.37	68.17±3.37	74.60±3.68
	after	58.07±2.86	58.80±2.94	61.40±3.17	64.50±3.29	59.60±3.95
	decrease	21.35±1.03	13.31±0.63	7.95±0.36	5.38±0.22	20.11±0.95
aeration porosity %	before	29.31±1.35	28.12±1.31	25.44±1.22	24.22±1.19	23.10±1.12
	after	52.11±2.33	54.21±2.34	51.91±2.19	44.22±2.16	42.18±2.02
	increase	77.79±3.82	92.78±4.55	104.05±5.07	82.58±3.92	82.60±3.85
Water-holding porosity %	before	44.52±2.21	39.71±1.94	41.26±1.99	43.95±2.08	51.50±2.42
	after	5.96±0.22	4.60±0.21	9.49±0.42	8.30±0.37	17.41±0.83
	decrease	86.61±4.25	88.42±4.31	77.00±3.68	81.11±3.94	66.19±3.20
pH	before	7.43±0.29	7.38±0.24	7.03±0.26	7.34±0.27	7.20±0.23
	after	7.32±0.34	7.45±0.36	7.65±0.37	7.48±0.36	7.61±0.37
	increase%	-1.48±0.067	0.95±0.041	8.82±0.41	1.91±0.094	5.69±0.26
EC ($ms\cdot cm^{-1}$)	before	0.15±0.002	0.15±0.002	0.20±0.002	0.12±0.003	0.12±0.003
	after	0.46±0.023	0.22±0.012	0.24±0.007	0.18±0.005	0.24±0.002
	increase%	206.66±2.0	46.00±2.3	20.00±1.4	50.00±2.3	50.00±1.33

Table 5 Changes in the nutrient contents of compound substrates after seedling cultivation

group		A	B	C	D	E
organic matters %	before	54.33±2.72	50.90±2.53	43.00±2.13	37.00±1.82	61.50±3.06
	after	9.45±0.46	19.14±0.94	14.43±0.73	9.93±0.48	15.76±0.77
	decrease	82.61±5.85	62.40±6.82	66.44±6.65	73.16±6.22	74.37±3.51
N%	before	0.86±0.041	0.93±0.042	2.29±0.09	1.38±0.065	0.85±0.039
	after	0.49±0.022	0.51±0.023	0.68±0.032	0.65±0.031	0.24±0.011
	decrease	43.02±1.98	45.16±2.04	70.31±3.32	52.90±2.39	71.76±3.41
P_2O_5%	before	0.36±0.017	0.56±0.026	0.90±0.042	0.59±0.027	0.62±0.029
	after	0.095±0.0045	0.16±0.0079	0.58±0.025	0.22±0.0099	0.069±0.0032
	decrease	73.61±3.33	71.43±3.21	35.56±1.56	62.71±2.71	88.87±4.03
K_2O%	before	1.47±0.074	1.68±0.082	2.13±0.097	1.43±0.068	0.75±0.035
	after	0.59±0.027	0.72±0.034	0.63±0.031	0.61±0.029	0.43±0.021
	decrease	59.86±2.86	57.14±2.74	70.42±3.43	57.34±2.73	42.67±1.87
Total nutrient %	before	2.69±0.12	3.17±0.14	5.32±0.25	3.40±0.18	2.22±0.12
	after	1.18±0.06	1.40±0.07	1.89±0.09	1.47±0.07	0.75±0.03
	decrease	56.13±6.32	55.84±6.62	64.47±6.39	56.76±7.35	66.22±7.21

Table 6 Morphologies of seedlings raised on different substrates

Treatment		A	B	C	D	E
survival rate %		99	99.5	100	97.5	99.5
height/cm	average	20.0±0.95	27.2±1.33	31.4±1.55	29.6±1.47	22.2±1.08
	Maximum	44.0±2.10	51.0±2.46	63.0±3.05	47.0±2.23	38.0±1.85
Ground	average	2.8±0.13	3.7±0.17	4.1±0.22	3.9±0.18	3.3±0.15

diameter/mm	Maximum	3.9±0.18	4.7±0.22	5.5±0.26	5.1±0.23	4.4±0.21
total root length/cm		2169.79±107.96	4377.77±215.32	2161.92±107.21	2864.55±142.53	3900.97±192.67
Root surface area/cm^2		371.30±18.23	768.81±38.23	397.98±19.56	596.43±29.62	683.89±34.01
Average root diameter/mm		0.56±0.026	0.56±0.027	0.58±0.027	0.66±0.032	0.56±0.025
Root volume cm^3		5.10±0.24	10.86±0.52	5.90±0.30	10.01±0.51	9.58±0.45

Table 7 Pearson correlation analysis of physical and chemical properties of substrate and seedling morphology

	Ground diameter	Seedling height	Total root length	Root surface area	Moisture content	Total porosity before seedling cultivation	Total aeration porosity before seedling cultivation	Organic matter content before seedling cultivation	Total nutrient content before seedling cultivation	Total nutrient after seedling cultivation	Change in organic matter content	Change in total nutrient content
Ground diameter	1	0.982**	0.044	0.181	-0.972**	-0.891*	-0.438	-0.723	0.754	0.723	-0.905*	0.732
Seedling height		1	-0.094	0.049	-0.997**	-0.944*	-0.311	-0.823	0.820	0.830	-0.957*	0.773
Total root length			1	0.971**	0.080	0.059	-0.166	0.412	-0.497	-0.471	0.087	-0.486
Root surface area				1	-0.055	-0.055	-0.282	0.219	-0.447	-0.393	-0.074	-0.452
Moisture content					1	0.967**	0.238	0.818	-0.826	-0.853	0.966**	-0.771
Total porosity before seedling cultivation						1	-0.005	0.100	-0.039	0.176	0.130	-0.148
Total aeration porosity before seedling cultivation							1	0.838	-0.814	-0.937*	0.952*	-0.710
Organic matter content before seedling cultivation								1	-0.669	-0.810	0.906*	-0.562
Total nutrient content before seedling cultivation									1	0.935*	-0.728	0.983**
Total nutrient after seedling cultivation										1	-0.841	0.855
Change in organic matter content											1	-0.633
Change in total nutrient content												1

*- Correlated at the significant level of 0.05 (bilateral)

**-Correlated at the significant level of 0.01(bilateral)

Figure 1 Temperature changes of the four compost piles during composting

585

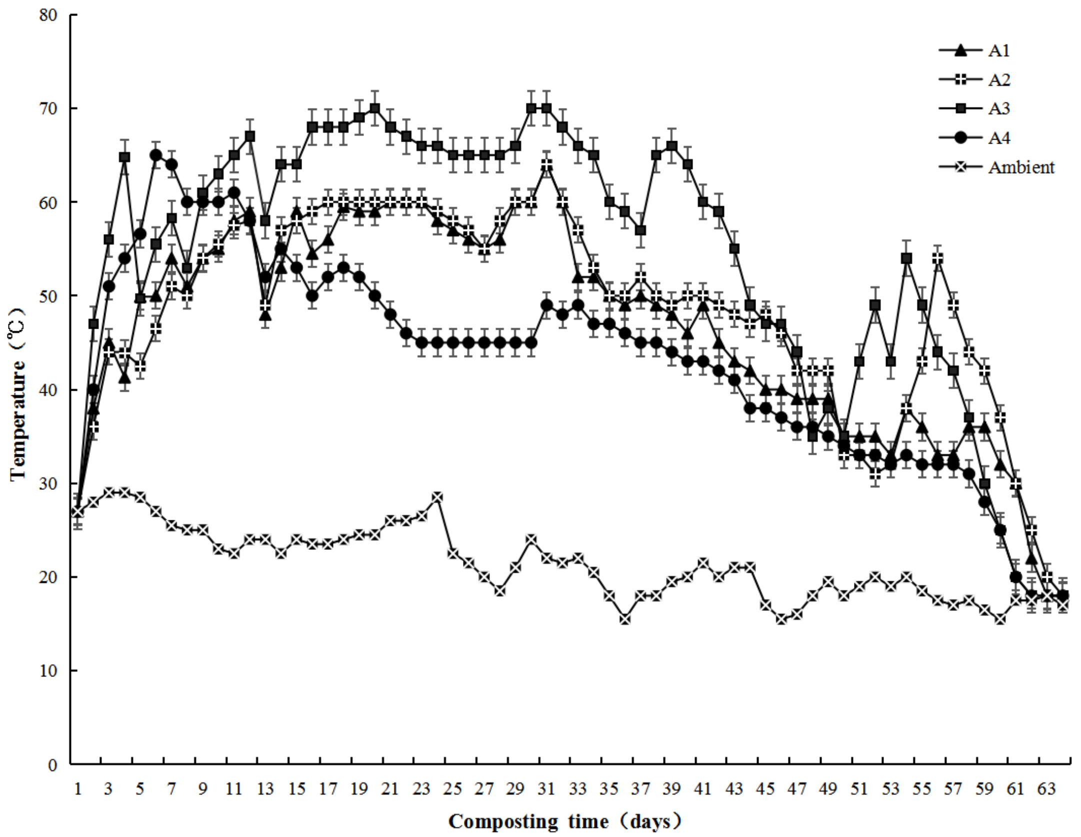

A1
A2
A3
A4
Ambient
Temperature（℃）
Composting time（days）
0
10
20
30
40
50
60
70
80
1
3
5
7
9
11
13
15
17
19
21
23
25
27
29
31
33
35
37
39
41
43
45
47
49
51
53
55
57
59
61
63

 Click here to access/download;Figure;Fig 1.tif

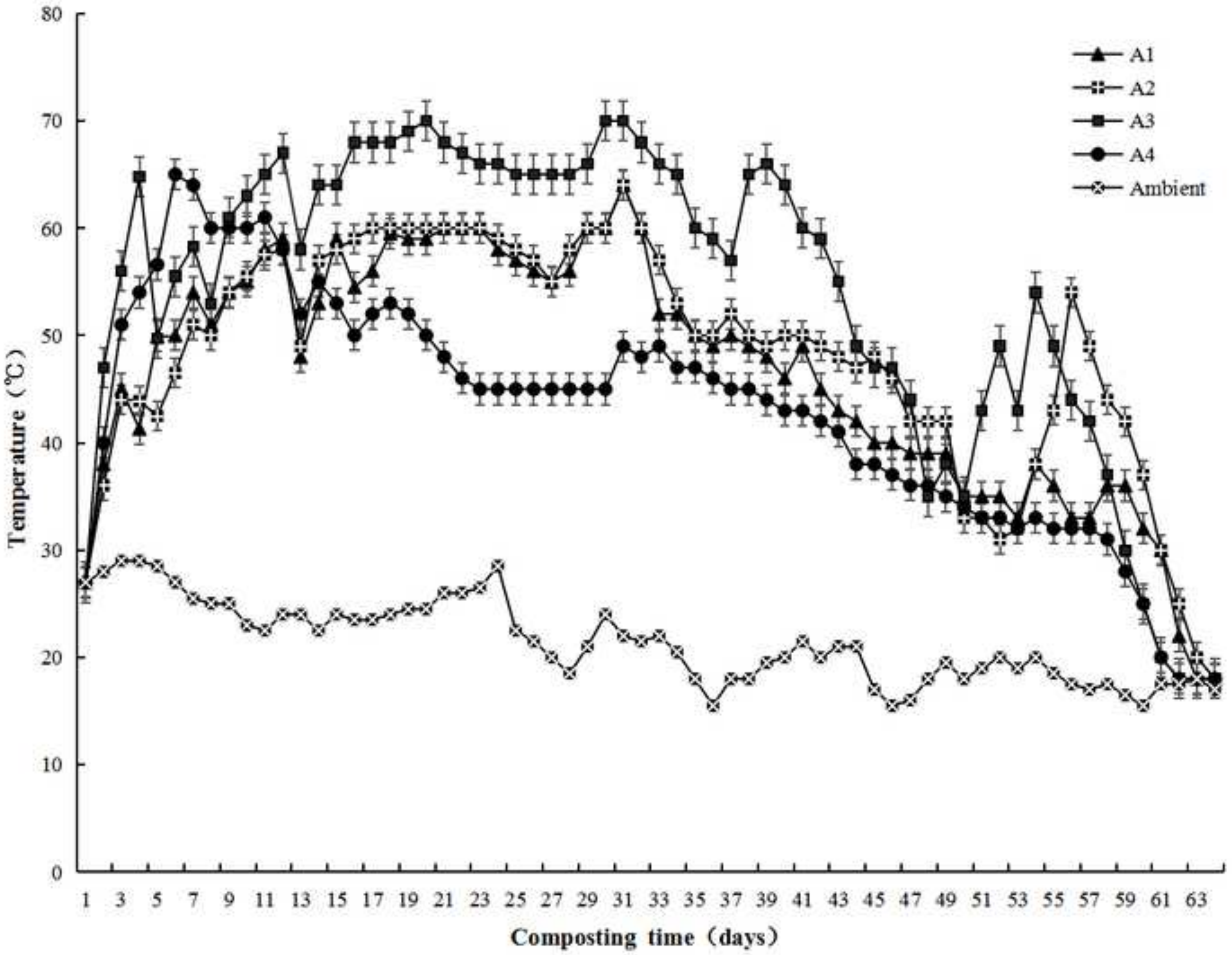

Formulation of substrates with agricultural and forestry wastes for Camellia oleifera Abel seedling cultivation

~~Nianjin Wang~~[~~1¶~~]~~,~~Fei Zhou[~~2~~1¶],Nianjin Wang[2¶],Jinping Zhang[~~2~~1*],Xiaohua Yao[~~2~~1],Tiantian Zhang[1],Xiaofeng Zhang[3]， Lingyan Zhan[4],Jieman Li[4]

[1]Research Institue of subtropical Forestry, Chinese Academy of Forestry, Hangzhou, Zhejiang, China~~Chun 'an County Forestry Bureau,Hangzhou, Zhejiang, China~~

[2]Chun 'an County Forestry Bureau,Hangzhou, Zhejiang, China~~Research Institue of subtropical Forestry, Chinese Academy of Forestry, Hangzhou, Zhejiang, China~~

[3]Fuyang District Environmental Protection Bureau, Hangzhou , Zhejiang, China

[4]Taihu County Huayuan Agricultural Science and Technology Development Co. LTD,Anqing,Anhui, China

* jinpingzhang@126.com

Formatted: Superscript

Abstracts： Five Camellia oleifera Abel seedling ~~compound~~ substrates ~~for the cultivation of Camellia oleifera Abel seedlings~~ were prepared using the ~~obtained by substituting the peats in~~ conventional ~~substrate~~ formula, but~~s~~ with the peat substituted by the composts of Camellia oleifera shell, pine chips, palm fiber residues, chicken manure, and sheep manure. The ~~changes in the~~ physical and chemical properties of the prepared substrates before and after seedling cultivation were determined and their effects ~~of these substrates~~ on the ~~development~~ growth of Camellia oleifera seedling were analyzed. It was found that the survival rates of the one-year-old seedlings produced from stem cuttings ~~in~~ on all substrates ~~all groups~~ at 6 months were greater than 97.5%~~ after 6 months cultivation~~. As compared with the conventional substrate, ~~The~~ the substrates formulated with the composts were able to promote the seedling growth upon ~~seedlings exhibited greater seedling~~ heights, ~~larger~~ ground diameters, ~~longer~~ root ~~system~~lengths and ~~larger total~~ root volume~~ than those obtained with conventional substrates~~. The ~~compound~~ substrate ~~formulated~~ prepared with the compost of Camellia oleifera shell+ palm fiber residue+ chicken manure (A3), vermiculite and perlite (6:3:1) was~~is~~ the most optimal, which ga~~i~~ve~~s~~ ~~the~~ 100% seedling survival rate~~ of cultivated seedlings~~, the greatest seedling height~~s~~, and the largest ground diameter~~s~~. In particular, the ground diameters and 26.67% of the seedling heights reach the grade 1 standard for ~~the~~ two-year-old seedlings.

Key words： Cultivation substrate; ~~；~~ ~~compound~~ substrate; ~~；~~ agricultural and forestry waste; ~~；~~ C~~c~~amellia oleifera; ~~；~~ physical and chemical properties

Formatted: Font: (Default) Times New Roman

1. Introduction

In China, 2 billion tons of a variety of agricultural and forestry wastes are produced every year. These wastes are renewable and biodegradable biomasses, yet only a small

portion ~~are~~ is reused. The majorit~~yies~~ of them are incinerated, buried, or disposed randomly, which is the waste of resources and causes environmental pollutions (Guo et al., 2021; Zhao et al., 2021; Lu et al., 2021). Agricultural and forestry wastes can be reutilized as the cultivation substrates for flowers, seedlings, and vegetables after treated by high-temperature aerobic fermentation (Zhao et al., 2021). Such applications not only re-utilize biomass wastes and reduce environmental pollutions, but also effectively improve the ecological environment (Nie, 2017) and realize carbon sequestration.

Camellia oleifera Abel. is an important woody oil tree species in southern China (Zhuang, 2008). The rapid development of tea tree oil processing industry has ~~been~~ dramatically increased~~ing~~ the production of by-products, such as Camellia oleifera shells. Composting ~~of~~ these by-products can not only prevent ~~the~~ wasting~~e~~ ~~of~~ resources and environmental pollution, but also provide ~~plant cultivation~~ substrates and organic fertilizers to improve soil quality and inhibit ~~the occurrence of~~ soil-borne diseases. ~~Seed box~~Pot method with light substrates shows great advantages, such as all-season operation, high survival rates, no slow growth period, fast growth, less root damage during transplanting, long afforestation seasons, and low transportation costs for seedling production. (Wang et al., 2018; Liu et al., 2019; Lv et al., 2017; Yang et al., 2018). Substrate ~~selection~~ is one of the key factors affecting the performance of ~~seed box~~pot production of seedling (She et al., 2020). The ~~The c~~conventional ~~camellia~~ Camellia oleifera seedling ~~production is mostly based on the~~ substrates are formulated with topsoil and peat. Peat is a non-renewable resource, and its ~~efficiency for~~performance for Camellia oleifera seedling production can be further improved (Wu et al., 2017). ~~Therefore, s~~Substituting peat with agricultural and forestry wastes has become a research hotspot. For example, Lu et al. reported the Camellia oleifera seedling production ~~on~~ using the substrate formulated with the compost of forest wastes, weeds, and vermicompost (Lu et al., 2021). Wu et al. successfully obtained Camellia oleifera seedlings with the substrate containing the compost of sawdust, decomposed litter, biogas residue and yellow soil (Wu et al., 2017). Dai et al. also formulated the substrate with the compost of bagasse, cassava skin, peanut shells, charcoal ~~ash~~ash, and garden soil ~~for the production of~~to produce Camellia oleifera seedlings (Dai et al., 2016). All these reports used the composts of agricultural and forestry wastes ~~as~~ as the substrates for the seedling production of Camellia oleifera, yet lacked the detailed information of the ~~sources of the~~ raw material sources~~s~~, composting formula and composting method. ~~In addition, t~~The variation in the ~~sources of~~ substrate raw material sources makes it difficult to obtain ~~the~~ consistent composts. The quality of ~~the compost of~~ Camellia oleifera shell compost has been successfully improved by adding different nitrogen sources, such as urea, compound fertilizer, and pig manure and EM bacteria (Luo, 2011), but no application research on the composts has been carried out.

Composting is a major ~~processing~~ process of recycling agricultural and forestry wastes. It is generally divided into aerobic composting and anaerobic composting. Aerobic composting decomposes organic matters under aerobic conditions mostly into CO_2 and water and releases heat. Anaerobic composting mainly produces methane,

~~CO_2~~CO_2, and low molecular weight intermediates, such as organic acids, etc. Most conventional composting is conducted under anaerobic conditions, which tends to release odors (Li et al., 2017). The modern composting processes are~~is~~ mainly conducted under aerobic conditions with controlled water contents, C/N ratios and ventilation. Aerobic composting can transform unstable agricultural and forestry wastes into stable substances, during which pathogenic bacteria are killed. The composts~~ing~~ thus can be safely handled and stored. They are also a good seedling substrate, soil conditioner and organic fertilizer (Zhang et al., 2021).

In this work, aerobic composting was conducted with~~using~~ C~~c~~amellia oleifera shells, pine chips, palm fiber residues, chicken manure and sheep manure as the main~~jor~~ raw materials using different formulas. The obtained composts were respectively mixed with boiler slag, vermiculite, and perlite at certain ratios as the substrates to grow ~~for the cultivation of C~~Camellia oleifera seedlings. The changes in the physical and chemical properties of the substrates after seedling cultivation and the relationship between composting raw materials and the seedling development were comprehensively evaluated and analyzed, aimed to optimize a composting formula and substrate formula for the cultivation of Camellia oleifera seedling~~s~~ and provide ~~the research~~ experimental data and a scientific reference for the recycling and composting of agricultural and forestry wastes including ~~, such as~~ C~~c~~amellia oleifera shells~~,~~ as the substrate exclusively for the ~~seed box~~pot cultivation of Camellia oleifera seedlings.

2. Materials and methods

2.1 Compost materials and preparations

Camellia oleifera shells, palm fiber residues, chicken manure, pine chips, and sheep manure were provided by Anhui Wanxiuyuan Ecological Agriculture Group Co., Ltd., (Anqing, Anhui Province, China). The Camellia oleifera shells and pine chips were smashed to the sizes of 5-8 mm before use. The compost bacteria were purchased from Yijiayi Biological Engineering Co., Ltd. (Zhengzhou, China) and urea was obtained from Henan Jinkai Chemical Investment Holding Group Co., Ltd (Zhengzhou, China). The primary properties of the compost raw materials are listed in Table 1.

Table 1 Primary properties of compost raw materials

~~**Table 1 Primary properties of compost raw materials**Table 1 Primary properties of compost raw materials~~

~~Raw material/Property~~	~~TOC (%)~~	~~TN (%)~~	~~C/N~~	~~Cellulose/%~~	~~Hemicellulose/%~~	~~Lignin/%~~
~~Camellia oleifera shells~~	~~51.80±2.58~~	~~0.729±0.03~~	~~71.359±3.74~~	~~18.62±0.22~~	~~49.34±0.07~~	~~29.71±0.14~~
~~Brown silk chips~~	~~46.80±2.23~~	~~2.68±0.14~~	~~17.557±1.76~~	~~28.15±0.18~~	~~20.58±0.08~~	~~44.08±0.16~~
~~Pine chips~~	~~54.20±2.74~~	~~0.285±0.01~~	~~190.964±5.19~~	~~48.36±0.11~~	~~27.59±0.12~~	~~19.58±0.21~~
~~Chicken mature~~	~~34.39±1.75~~	~~2.73±0.14~~	~~12.667±1.31~~	-	-	-
~~Sheep mature~~	~~36.47±1.81~~	~~2.78±0.14~~	~~13.186±1.32~~	-	-	-

Formatted: Font: 7.5 pt, Bold

Formatted Table

~~Urea~~	~~20±0.18~~	~~46.4±1.22~~	~~43.1±1.12~~	-	-	-

~~“-”indicates extremely low content or none~~

2.2 Composting process

Composting was conducted by window composting in the composting plant of Anhui Wanxiuyuan Ecological Agriculture Group Co., Ltd. from August to November 2020 ~~by the windows composting system~~. Four composting ~~groups~~ piles including A1: Camellia oleifera shell + urea (C/N 60); A2: Camellia oleifera shell+ sheep manure (C/N 55); A3: Camellia oleifera shell+ palm fiber residue + chicken manure (C/N 55) /N 30); and A4: pine chips+ chicken manure (C/N 40.7) were prepared, ~~mixed~~mixed, and stirred evenly. The moisture content of each pile was adjusted to 55-60% and 0.1% fermentation bacteria were added into each pile. Each pile was then thoroughly mixed, stirred evenly, and stacked for high-temperature aerobic fermentation at the same time. The composting piles were turned for aeration and sampled by the five-point sampling method every 7 days. For each sample, half portion was stored at -20℃ and the other half was dried at 65℃ and ground.

2.3 Compound substrate preparation and seedling ~~nursing~~cultivation

Seedlings ~~nursing was~~were cultivated ~~carried out~~ at the nursery of Anhui Wanxiuyuan Ecological Agriculture Group Co., Ltd. located in Taihu County, Anqing, Anhui Province between 30°09' N to 30°46' N and 115°45 'E to 116°30 'E. The temperatures during the ~~nursing~~ experiment varied between from -6 °C and 34 °C, and there were 24 days with temperatures lower than 0 °C ~~lasted for 24 days~~.

~~The c~~Compound substrates were prepared with the composts obtained above and inorganic matrices using the formulas shown in Table 2. In December 2020, one year old Camellia oleifera seedlings with intact root systems, the heights of 13 ± 3 cm and ground diameters of 0.22 ± 0.02 cm were respectively transplanted into ~~seed boxes~~pots with the diameter of 16 cm and height of 16 cm. The ~~boxes~~ pots were filled with the compound substrates listed in Table 2. For each substrate, 200 seedlings were planted and ~~replicates were prepared and~~ allowed to grow for 6 months. The substrates were then sampled and analyzed for physical and chemical properties~~analysis~~. During the seedling cultivation, ~~,~~ the substrates were maintained moist, and watered thoroughly when ~~the substrate~~they became dry during the fast growing period.

Table 2 Formulas of compound substrates

~~**Table 2 Formulas of compound substrates**~~~~Table 23 Formulas of compound substrates~~

2.4 Analyticals ~~is~~ methods

Formatted: Font: 12 pt

Formatted: Font: 12 pt

During composting, the compost temperature was measured from~~in~~ the upper (10 cm from the top), middle and lower (10 cm from the bottom) parts of the pile at around 3:00 pm every day, and the ambient temperature of the day was recorded at the same time. The bulk density, total porosity, aeration porosity, water-holding porosity, electrical conductivity (EC) and pH were also measured following the Chinese Forestry Industry Standard GB/T 33891-2017 of organic substrates for greening. Total organic carbon (TOC), total nitrogen (TN), total phosphorus (TP), total potassium (TK) and germination index (GI) were determined by the method of Zhang et al. (2018). The organic matter content, total nutrient content, C/N ratio and survival rate of Camellia oleifera seedling~~s~~ were calculated with Eq. (1), (2), (3) and (4), respectively.

Organic matter (%) = TOC (%) × 1.724 (1)

Total nutrient (%) = TN (%) + P_2O_5 (%) + K_2O (%) (2)

C/N=TOC(%)/TN(%) (3)

Survival rate = number of survived seedlings ÷ total number of ~~cultivated~~ planted ~~camellia~~ seedlings × 100% (4)

After 185 days of cultivation, 60 seedlings ~~in each group~~ were randomly collected from each group and measured for ground diameter~~s~~ and height~~s~~. Five seedlings were randomly selected from each group and analyzed for ~~the~~ root length~~s~~, surface area~~s~~, volume~~s~~ and average diameter~~s~~ ~~of their root systems~~ using a Wanshen LA-S root system analyzer to evaluate the effects of the physical and chemical properties of substrate on the growth of Camellia oleifera seedling. The obtained data were processed and analyzed using the EXCEL and SPSS software~~s~~.

3. Results and discussion

3.1 Compost temperature

Temperature is one of the important indicators to evaluate the compost maturity (Gu et al., 2015; Bustamante et al., 2008). ~~The~~ A composting process is generally divided into four phases, e.g., the mesophilic phase, the thermophilic phase (>50 °C), the cooling phase, and the maturation phase. Pathogenic microorganisms, insect eggs and weed seeds ~~can be~~are killed at the thermophilic phase when the compost temperature~~s~~ increases to over 55 ℃, and thus compost becomes ~~producing h~~harmless ~~composts~~. The fermentation bacteria are rapidly deactivated as temperature increased to over 63 ℃, and the compost tempeture drops, leading to ~~temperature drops of the compost~~cooling phase (Bernal et al. 2009). As can be seen from Figure 1, the temperatures of all compost~~ing~~ piles are much higher than the ambient temperature during the composting, indicating that ~~the~~ ambient temperature has little effects on the composting. The initial temperatures of ~~the 4 groups of~~all composting piles were 27±0.6 ℃. The temperatures of A1, ~~of~~ A2, A3 and A4 ~~composting piles~~ increased to above 50℃ on the day 6, 7, 3 and 3, respectively, and remained at 50 ℃ for 20, 23, 39, and 9 days, and at 60 °C for 4, 7, 22, and 6 days, respectively, ~~complying~~ conforming to the safety and hygiene requirements (Wang et al., 2017). The four compost~~ing~~ piles

entered the cooling phase on day 33, 34, 44 and 15, respectively and their maturation phases lasted 38, 42, 44 and 21 days, respectively. The sudden temperature drops are due to heat dissipation during manual turning for aeration (Qian et al, 2014; Guardia et al, 2010a).

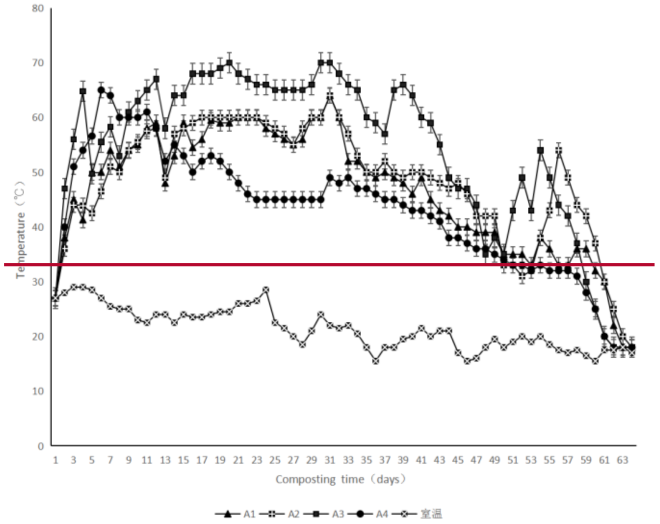

~~Figure 1 Temperature changes of the four composting piles during composting~~

Figure 1 Temperature changes of the four compost piles during composting

The~~ A3~~ compost~~ing~~ pile A3 exhibited the largest heating rate, the highest high temperature, and the longest thermophilic phase. It can be explained that, first, the initial C/N (30) of A3 is most suitable for microbial reproduction (Bernal et al. 2009). Second, there is a large amount of chicken manure in the pile~~A3~~. Chicken manure is rich in protein and urea that can be easily decomposed and utilized by microorganisms. Third, the palm fiber residues can improve the aeration porosity of the compost, which is conducive to the proliferation of the microorganisms and heat generation. ~~Groups~~ Piles A1, A2 and A3 all contain C~~c~~amellia oleifera shells, but have different C/N ratios due to different excipients. The C/N ratio of A3 is 30, which is in the optimal range ~~of~~ (25-35), while those of A1 and A2 are 60 and 55, respectively, that ~~suggesting~~ indicate ~~insufficient~~ low nitrogen contents. The activity of microorganisms is lower under the insufficient nitrogen conditions, and thus ~~which slows down~~ the composting reactions are slow (Ma et al., 2020). The temperature of pile A4 increased rapidly and thus its initial temperature was higher. However, it entered the cooling phase earliest and its~~the~~ temperature dropped fastest. It can be explained that ~~The~~ the nutrients in the chicken manure are rapidly ~~first~~ decomposed~~ rapidly~~. The main components in pine chip are cellulose and lignin that are difficult to be degraded and the hemicellulose content ~~in the pine chip~~ is only 27.59%~~, and the majority is cellulose and lignin that are difficult~~

to be degraded. In contrast, the Camellia oleifera shellsss in other three groups are rich in easily highly degradable hemicellulose (49.34%), which can prolong contributes to the longthe thermophilic phases. These results suggest that the physical and chemical properties of raw materials – directly decide affect the composting process and the physical and chemical properties of compost (Table 3).

3.2 *Physical and chemical properties of composts and substrates*

The composts of agricultural and forestry wastes can be used alone as a seedling substrate or can be formulated with other matrices to form differentas seedling substrates. The substrates with large bulk densities are favorable for the seedling handling. The cohesions of the substrates with small bulk densities are poor, which is inconducive to the root fixation of root system (Li et al., 2020). The bulk densities of A1, A2, A3, and A4 composts are 0.39 $g\cdot cm^{-3}$, 0.40 $g\cdot cm^{-3}$, 0.47$g\cdot cm^{-3}$, and 0.42 $g\cdot cm^{-3}$, all within the ideal bulk density range of 0.1-0.8 $g\cdot cm^{-3}$ (Wang et al., 2021). Porosity is another important physical property that affects the aeration, drainage and water holding functions capacity of a substrate. The total porosities of A1, A2 and A3 composts are 73.94%, 74.13% and 65.90%, respectively, which are all within the ideal total porosity ramgerange of seedling substrate (40%-75%) (Wu, 2006; Wang et al., 2013). The total porosity of A4 compost (is 83.4%) is ,too high as a seedling substrate and can which needs to be adjusted with other matrices. All tThe pHs, seed germination indexes (GIs), and organic matter contents of the composts are in the ranges of 7-8, 89.12-99.42, and 65.86-81.55%, respectively, all of which meet the standard NY/T525-2021 for organic fertilizer defined by the Ministry of Agriculture and Rural Affairs of the People''s Republic of China (pH 5.5-8.5, GI≥70%, and organic matter content≥30%). Germination index (GI) is a parameter that can be used to quickly evaluate the phytotoxicity of a compost in a short period of time and is of significant great importance in actual productions. The GIs of the 4all groups of composts are >85%, and thus the composts can be considered non-toxic to plants (Yamamoto, N. et al. 2011). The total nutrient contents of A1, A2, A3, and A4 composts are measured to be 3.27%, 3.85%, 6.32%, and 1.80%, respectively. Thate total nutrient content of A3 compost reaches the standard NY/T525-2021 for organic fertilizer (≥4%) and thus it can be used as an organic fertilizer or a seedling substrate raw materialcomponent. The rest 3 groups can be used as cultivation substrates or substrate raw materials.

Table 3 Physical and chemical properties of the composts of agricultural and forest waste and other components for the preparation of compound substrates

Table 3 Physical and chemical properties of the composts of agricultural and forest waste and other components for the preparation of compound substrates

3.3 *Changes in physical and chemical properties of compound substrates after seedling cultivation*

The composts of agricultural and forestry wastes have relatively poor The water holding capacities of the obtained composts are low, and thus vermiculite with a better

water holding ability is introduced. The raw materials listed in Table 3 ~~are~~ were mixed at the ratios listed in Table 2, and the physical and chemical properties of the corresponding compound substrates ~~are~~ were measured ~~shown in~~(~~-~~Table 4). The bulk densities of compound substrates A, B, C, D, and E ~~are~~ were measured to be 0.41 g·cm^{-3}, 0.47 g·cm^{-3}, 0.41 g·cm^{-3}, 0.42 g·cm^{-3}, and 0.41 g·cm^{-3}, respectively and their total porosities ~~are~~ were 73.83%, 67.83%, 66.70%, 68.17% and 74.60%, respectively, all within the ideal ranges of 0.1-0.8 g·cm^{-3} and of 40%-75% (Wang et al., 2021 ; Wu, 2006; Wang et al., 2013). Their aeration porosities (29.31%, 28.12%, 25.44%, 24.22% and 23.10%), pH (7.03-7.43) and EC values (0.12-0.20 ms·cm^{-1}) all conform to the national standard "Organic Substrates for Greening" (GB/T 33891-2017) of the People's Republic of China ~~of "Organic Substrates for Greening" (GB/T 33891-2017)~~ with the requirements of total porosity≥20%, pH 5.0-7.6 and EC≤0.65 ms·cm^{-1}. ~~All the~~Yet none of the pHs ~~do not~~ conforms to the Forestry Industry Standard ~~of the People's Republic of China~~ LYT 2314-2014 "Technical Regulations for Seed Box Production of Camellia Container Seedling" of the People's Republic of China (pH 5.0-6.5).

~~Table 4 and Table 5~~ The changes in ~~compare~~ the physical and chemical properties of the five compound substrates after ~~before and after~~ the seedling cultivation are summarized in Table 4 and 5. As can be seen, the bulk densities, aeration porosities and EC values of all groups increase, while their total porosities and water-holding porosities, organic matter contents and total nutrient contents decrease after ~~6 months~~the 6-month cultivation. It can be explained that, first, most of the hemicellulose in the compost raw material ~~wastes~~ is degraded during composting (Zhang et al., 2019), and ~~the~~ large amounts of refractory cellulose and lignin continue to decompose slowly during the cultivation, which increases the aeration porosity. Second, with the seedling growth and development ~~of the seedlings~~, the root elongation squeezes the substrate and thus decreases the total porosity and water-holding porosity. Third, the seedling growth ~~growth of seedlings~~ consumes large amounts of organic matters, nutrients, and minerals, which decreases the contents of organic matters, nitrogen, P_2O_5, K_2O and total nutrient~~s~~. The seedling development ~~of the seedlings~~ also accelerates the decomposition of ~~the~~ refractory substances, resulting in ~~the increases in~~increased EC value~~s~~. The seedlings of group C show the highest survival rate, the greatest seedling ~~height~~height, and the largest ground diameter (Table 6), and the aeration porosity of the substrate is increased by up to 104.05%. The total porosities of substrates A, B, C, D and E decrease by 21.35%, 13.31%, 7.95%, 5.38% and 20.11%, respectively, after the cultivation. Both substrates C and D contain the palm fiber residue~~s~~ ~~with~~ a high lignin content~~s~~ (Table 1). ~~The l~~Lignin is difficult to be degraded, which explains the smaller changes in the total porosity of two~~the~~ substrate~~s~~. Therefore, it can be concluded that the substrate porosity ~~change~~changes ~~of substate~~ after seedling cultivation ~~is~~ are related to the cellulose and lignin contents of the compost feedstock. Substrates A, B, C and D all contain the Camellia oleifera shell~~s~~ with a high hemicellulose content~~s~~ (Table 1). The compost in substrate A was abstained in the absence of livestock ~~and~~ or poultry manure and the microbial abundance (Jinping et al. 2021) and temperature of the compost were lower than those of the compost in substrate B (Figure 1). Therefore, the hemicellulose

degradation rate of substrate A is lower than that of substrate B (Zhang et al. 2021). The ~~degradation of~~ hemicellulose in substrate A was~~is~~ further degraded ~~continued~~ during the seedling cultivation~~ of seedlings~~, ~~resulting in~~leading to the largest decrease in total porosity. The cellulose content of pine chips in substrate E is high (Table 1). Cellulose~~,~~ ~~which~~ is ~~more~~ difficult to be decomposed, but easier than lignin. Therefore, small amounts of hemicellulose, cellulose and lignin continue to decompose during the seedling cultivation, which causes ~~leading to~~ the significant decreases in the total porosity of substrate E.

Table 4 Changes in the physical and chemical properties of compound substrates after seedling cultivation

Table 5 Changes in the nutrient contents of compound substrates after seedling cultivation

~~**Table 4 Changes in the physical and chemical properties of compound substrates after seedling cultivation**~~

~~**Table 5 Changes in the nutrient contents of compound substrates after seedling cultivation**~~

3.4 *Relationship between physical and chemical properties of compound substrate and seedling morphology*

Survival rate is an important indicator for evaluating the successful cultivation of ~~camellia oleifera~~ seedlings. The seedling survival rates ~~of the seedlings~~ of all groups ~~A, B, C, D and E~~ are ~~99%, 99.5%, 100%, 97.5%, and 99.5%, respectively (Table 6), which are all~~ above 97% (Table 6), ~~and~~ significantly higher than that (42.50-67.50%) obtained using the compost of rice hulls, bark, and sawdust (rice hulls 20%-26%, bark 28%-44%, sawdust 36%-52%) as the substrate (Lu et al., 2017).

Seedling height and ground diameter are important indicators ~~for measuring the~~of seedling quality ~~of seedlings~~ (Wang et al., 2021). As shown in Table 6, the average heights and maximum heights of the seedlings cultivated on the 5 substrates for 6 months are in the order of C> D>B>E>A and C>B>D>A>E, respectively. Both of their average ground diameters and ~~the~~ maximum ground diameters are in the order of C>D>B>E>A. The average ground diameter (4.1 mm) and average seedling height (31.4 cm) of ~~the seedlings in~~ group C respectively reach~~es~~ the first-grade and second-grade criteria~~, respectively,~~ defined in the standard GB/T 26907-2011 for two years old seedling (≥0.35 cm and ≥25 cm). ~~Among them~~In addition, 26.67% of the seedling heights reach the first-grade criteria (≥40 cm), and 45% of them reach the second-grade criteria. The average ground diameters of both groups B (3.7 mm) and D (3.9 mm) reach the first-grade criteria. Upon their ~~average~~ seedling heights~~s (27.2 cm and 29.6 cm)~~, 6.7% of group B and 15% of group D reach the first-grade criteria and the rest all reach the second-grade criteria. The average ground diameter (3.3 mm) of the seedlings in group~~of~~ E ~~group~~ meets the second-grade criteria (≥0.30 cm), but ~~and~~ only 28.3% of

Formatted: Font: Bold

Formatted: Space After: 0 pt

Formatted: Font: Bold

Formatted: Space After: 0 pt, Line spacing: single

Formatted: Font: 12 pt

the ~~average ~~seedling heights meet the second-grade criteria. Neither of the average ground diameter (2.8 mm) nor the average seedling height (20.0 cm) of the seedlings in group A conforms to the second-grade criteria, but 41.67% of the seedlings meet~~s~~ the second-grade standard for~~for~~ ground diameter and 15 % of them ~~seedlings ~~meet the second-grade standard for height. It is worth noting that the average seedling height of the seedlings in group C (31.4 cm) is 30.5 cm higher than that~~ose~~ of two-year-old C~~c~~amellia oleifera seedlings cultivated on the substrate prepared with coconut bran and yellow soil (coconut bran 75% +yellow soil 25%) ~~mixed with~~and 3 kg/m^3 of slow-release compound fertilizer from KOCH (USA) (Wang et al., 2018). The average seedling heights (31.4 cm, 29.6 cm) and average ground diameters (4.1 mm, 3.9 mm) of the seedlings in groups C and D are better than those (28.70 cm, 3.93 mm) of the seedlings raised on the substrate formulated with peat and the compost of rice hulls (peat 50% + (compost of rice hulls+ extruded perlite + vermiculite + boiler slag + imported slow-release fertilizer) 50%) for 18 months (Cui et al., 2017). The average seedling heights (27.2 cm, 31.4 cm and 29.6 cm) and average ground diameters (3.7 mm, 4.1 mm and 3.9 mm) of the seedlings in groups B, C and D are better than those (25.98 cm, 3.43 cm) of the seedlings raised on the substrate containing composted bagasse, cassava skin and charcoal ash (2:1:1) for 18 months (Dai et al., 2016).

The compound substrate C contains the highest amounts of N, P_2O_5, K_2O, and total nutrients before seedling cultivation, and the decreases in the contents of N, K_2O, and total nutrient and the increase in aeration porosity are the most significant after the seedling cultivation (Table 5 and 6). The seedling survival rate, height, and ground diameter of this group are also the ~~highest~~greatest. These results suggest that seedling height and ground diameter are mainly related to the total nutrient content ~~in seedling~~of the substrate, consistent with the results reported by Wu et al. and Zeng et al. (Zeng et al., 2021; Wu et al., 2018). ~~In addition, ~~Compared with those of other substrates, the N and K_2O contents of substrate C decrease most significantly, by 70.31% and 70.42% respectively, ~~which are the largest among those of the 5 substrates, ~~while the decrease in its P_2O_5 content is the smallest, suggesting that nitrogen and potassium can promote the growth and development of C~~c~~amellia oleifera seedling~~s~~ (Chen, 2009; Wang et al., 2010; Peng, 2019).

The organic matter contents of substrates A-E decreased by 82.61%, 62.40%, 66.44%, 73.16% and 74.37%, respectively, after the seedling cultivation due to the consumption by the seedlings and losses during watering. Before the seedling cultivation, the organic matter contents and total nutrient contents of~~in~~ the substrates are in the order of E>A>B>C>D, and C>D>B>A>E, respectively. The substrates with ~~a ~~high organic matter contents generally show~~has a ~~low total nutrient contents. The raw materials of the composts in substrates C and D are the same, but their formulas and ratios with other inorganic matrices are different. The ~~portion ~~ratio of the compost in substrate C is 20% higher than that in substrate D. Therefore, both the contents of organic matter and total nutrient of substrate C are higher than those of substrate D. The seedling heights and ground diameters of groups B, C, and D are significantly ~~higher~~ greater than those of the other two groups, but the decreases in ~~their ~~the organic matter

Formatted: Not Highlight

Formatted: Not Highlight

Formatted: Not Highlight

Formatted: Not Highlight

Formatted: Not Highlight

Formatted: Not Highlight

Formatted: Not Highlight

Formatted: Not Highlight

Formatted: Not Highlight

contents of their substrates are smaller, indicating that there are large amounts organic matters in the composts of Camellia oleifera shell and livestock/poultry manures that can be absorbed and utilized by the seedlings. The maximum decrease in the total nutrient, contents of substrate C is only 3.43%, and the average seedling height and ground diameter of the seedlings cultivated raised on it are the largest. Even though the organic matter content of substrate A is decreased by 82.61% after the seedling cultivation, it shows showed no significant advantages for the seedling growth of Camellia oleifera seedlings. It may be explained with the low degradation rate of lignocellulose during the composting process that results in low contents of the organic matters usable for the seedling development. All these results suggest that the height and ground diameter of Camellia oleifera seedlings raised on the substrates are closely related to the properties of composition of composting raw materials, composting formula, composting process and rate, and substrate formula. The Therefore, sstable composting raw materials, formula, composting process, and compound substrate formula are the keys factors to improvinge the quality and efficiency of the cultivation of Camellia oleifera seedling. cultivation.

Table 6 Morphologies of seedlings raised on different substrates

Table 6 Morphologies of seedlings raised on different substrates

Table 6 Morphologies of seedlings raised on different substrates

Treatment		A	B	C	D	E
survival rate %		99	99.5	100	97.5	99.5
height/cm	average	20.0±0.95	27.2±1.33	31.4±1.55	29.6±1.47	22.2±1.08
	Maximum	44.0±2.10	51.0±2.46	63.0±3.05	47.0±2.23	38.0±1.85
Ground diameter/mm	average	2.8±0.13	3.7±0.17	4.1±0.22	3.9±0.18	3.3±0.15
	Maximum	3.9±0.18	4.7±0.22	5.5±0.26	5.1±0.23	4.4±0.21
total root length/cm		2169.79±107.96	4377.77±215.32	2161.92±107.21	2864.55±142.53	3900.97±192.67
Root surface area/cm²		371.30±18.23	768.81±38.23	397.98±19.56	596.43±29.62	683.89±34.01
Average root diameter/mm		0.56±0.026	0.56±0.027	0.58±0.027	0.66±0.032	0.56±0.025
Root volume cm³		5.10±0.24	10.86±0.52	5.90±0.30	10.01±0.51	9.58±0.45

The physical and chemical properties of substrate also affect the morphological characteristics of Camellia oleifera seedling root system. The rRoot morphology can be characterized with the root growth indicators, such as root length, root surface area, root volume, and root diameter (Pang et al., 2018; Li et al., 2020). In general, the longer and thicker root systems with larger surface areas and volumes suggest better development and growth conditions. As shown in Table 6, the root morphologies of the seedlings raised on different substrates are significantly different. The seedlings of group B exhibitshowed the longest root system of 4377.77 cm, the largest root surface area of 768.81 cm^2, and the largest root volume of 10.86 cm^3, and both the root root surface area and volume of group A are the smallest. The root surface areas and root

Formatted: Not Highlight

Formatted: Not Highlight

Formatted: Not Highlight

volumes of the seedlings raised on the five substrates are in the orders of B>E>D>C>A and B>D>E>C>A, respectively. The average root diameter of group D is 0.66 cm, significantly larger than those of other groups (0.56 cm for groups A, B and E, and 0.58 cm for group C). Despite the ~~larger~~ larger root length, surface area and volume of group B, its average root diameter is similar to those of other groups. ~~Overall~~Yet~~,~~ the overall root development of group B is the best, possibly because the porosity and nutrient content of its~~the~~ substrate is more suitable for the root development of Camellia oleifera seedling. ~~Yet t~~The total root lengths, surface areas, and volumes of all five groups are better than those of the Camellia oleifera seedlings ~~cultivated~~ raised on ~~traditional~~ conventional substrates. For example, the seedlings grown on the substrate ~~, such as yellow soil, mushroom residue and peat substrate~~formulated with ~~(~~40% yellow soil + 15% pine forest topsoil + 20% mushroom residue + 20% peat + 5% manure~~)~~ for two years ~~which~~ only show the average total root length of 280.32 cm, the surface area of 106.72 cm^2, and the average root volume of 8.71 cm^3 (She et al., 2020).

3.5 *Correlation analysis of seedling ~~Morphology~~ morphology and substrate physical and chemical properties*

~~**Table 7 Pearson correlation analysis of physical and chemical properties of substrate and seedling morphology**~~

~~Table 7 Pearson correlation analysis of~~ seedling morphology and ~~physical and chemical properties of substrate and seedling morphology~~

	Ground diameter	Seedling height	Total root length	Root surface area	Moisture content	Total porosity before seedling cultivation	Total aeration porosity before seedling cultivation	Organic matter content before seedling cultivation	Total nutrient content before seedling cultivation	Total nutrient after seedling cultivation	Change in organic matter content	Change in total nutrient content
Ground diameter	1	0.982**	0.044	0.181	-0.972**	-0.891*	-0.438	-0.723	0.754	0.723	-0.905*	0.732
Seedling height		1	-0.094	0.049	-0.997**	-0.944*	-0.311	-0.823	0.820	0.830	-0.957*	0.773
Total root length			1	0.971**	0.080	0.059	-0.166	0.412	-0.497	-0.471	0.087	-0.486
Root surface area				1	-0.055	-0.055	-0.282	0.219	-0.447	-0.393	-0.074	-0.452
Moisture content					1	0.967**	0.238	0.818	-0.826	-0.853	0.966**	-0.771
Total porosity before seedling cultivation						1	-0.005	0.100	-0.039	0.176	0.130	-0.148
Total aeration porosity before seedling cultivation							1	0.838	-0.814	-0.937*	0.952*	-0.710
Organic matter content before seedling cultivation								1	-0.669	-0.810	0.906*	-0.562
Total nutrient content before seedling cultivation									1	0.935*	-0.728	0.983**
Total nutrient after seedling cultivation										1	-0.841	0.855
Change in organic matter content											1	-0.633

~~Change in total nutrient content~~	~~-~~

~~*- Correlated at the significant level of 0.05 (bilateral)~~
~~** Correlated at the significant level of 0.01(bilateral)~~

Table 7 Pearson correlation analysis of physical and chemical properties of substrate and seedling morphology

Seedling height, ground diameter and root activity are generally considered to be the most important indicators ~~for evaluating~~of seedling~~the~~ quality ~~of seedlings~~ (Lu et al., 2002; Ge et al., 2006). Herein, ~~We~~ we evaluated the Camellia oleifera seedling growth with ~~selected~~ seedling height, ground diameter and root morphology ~~to evaluate the growth of Camellia oleifera seedling~~ and analyzed the correlation between the physical and chemical properties of substrate and the seedling growth. As can be seen from Table 7, the moisture content of substrate is negatively correlated ~~with~~ to seeding ground diameter (r=-0.972, P<0.01) and seedling height (r=-0.997, P<0.01). Seedling height and ground diameter are positively correlated (r=0.982, P<0.01), and ~~the~~ root surface area and root length are positively correlated (r=0.971, P<0.01). The total porosity of substrate before seedling cultivation is negatively correlated ~~with~~ to seedling height (r=-0.891, P<0.05) and ground diameter (r=-0.944, P<0.05). The changes in the contents of organic matter and total nutrient ~~contents~~ of substrate after seedling cultivation reflect the amounts of substrate nutrients absorbed by the seedlings and the ~~the nutrient~~ losses of the substrate itself. The change in organic matter content is negatively ~~correlated with~~correlated to ground diameter (r=-0.905, P<0.05) and seedling height (r=-0.957, P<0.05), and is positively ~~correlated with~~correlated to moisture content (r=0.966, P<0.01), aeration porosity ~~before~~ seedling cultivation (r=0.952, P<0.05) and organic matter content before seedling cultivation (r=0.906, P<0.05). The change in total nutrient content is positively ~~correlated with~~correlated to the total nutrient content before seedling cultivation (r=0.983, P<0.01). The total nutrient content after seedling cultivation is negatively ~~correlated with~~correlated to the aeration porosity before seedling cultivation (r=0.937, P<0.05) and is positively ~~correlated with~~correlated to the total nutrient content before seedling cultivation (r=0.935, P<0.05). No correlation is found between the change in total nutrient content and seedling ground diameter (r=0.732) and seedling height (r=0.773). There are no significant correlations between the contents of organic matter and total nutrient ~~contents~~ before and after seedling cultivation and seedling ground diameter and height (r1=-0.723, r2=-0.823, r3=0.304, r4=184; r5=0.754, r6=0.820, r7=0.723, and r8=0.830). These results suggest that organic matters and nutrients that can be absorbed and utilized by Camellia oleifera seedling play a key role in the seedling growth and development.

4 Conclusion

Agricultural and forestry residues were composted using different formulas and the composting efficiencies were evaluated. It is found that the introduction of livestock

and poultry manures and the C/N ratios in the range of 25-35 can significantly increase the heating rate, prolong the thermophilic phase, and produce the composts with high total nutrient contents and GIs.

Five compound substrates were prepared using the formulas containing the obtained composts and inorganic substrates, vermiculite and perlite, with no peat added for the cultivation of one year old~~The annual~~ C~~c~~amellia oleifera ~~cutting~~ seedlings.~~s were cultivated after the composting products of agricultural and forestry residues were 100% substituted for peat and formulated with inorganic substrates including vermiculite and perlite.~~ The physical and chemical properties of the substrates before and after seedling cultivation and the seedling development were analyzed. The results show that, first, the physical and chemical properties of compost~~ing~~ raw materials, composting formula and degree of composting directly affect the physical and chemical properties of the obtained compost and corresponding compound substrate, as well as the ~~growth of~~ seedling growth. Second, the physical and chemical properties of compound substrate conform to the standards defined in GB/T 33891-2017 for seedling substrate, ~~-~~except that their pHs (7.03-7.43) do not meet the pH requirement (5.0-6.5) of the regulation LYT 2314-2014. However, the Camellia oleifera seedlings developed better on these substrates than on the traditional nursery substrates. Therefore, further study is needed to evaluate the effects of substrate pH on the growth of Camellia oleifera seedling. Third, the survival rates of the seedlings cultivated on the ~~all~~ five substrates for 185 day covering the severely hot summer and old winter are all greater than ~~≥~~97.5%~~ after 185 days cultivation covering the sever hot summer and cold winter~~. In particular, the seedlings cultivated on substrate C with the ~~that contains the~~ highest ~~amount of~~ total nutrient content ~~reaches~~ exhibit~~ed~~ 100% survival rate, and the largest seedling height and ground diameter. In addition, all of the~~the~~ seedling heights and 26.67% of ground diameters reach the first grade criteria for two years old seedling. Forth, the changes in the physical and chemical properties of substrate after seedling cultivation are correlated to~~directly affect~~ the growth and development of Camellia oleifera seedling. The initial total porosity is negatively correlated ~~with~~ to seedling height at the significant level, and the change in organic matter content is negatively ~~correlated with~~correlated to ground diameter and seedling height, and positively ~~correlated with~~correlated to the initial aeration porosity and initial organic matter content at significant levels. Based on these results, ~~It~~ it can~~is~~ ~~then~~ be concluded that the amounts of organic matters and ~~total~~ nutrients in the substrate that can be absorbed and utilized by ~~the~~ seedling play a key role in the seedling development.

~~Acknowledgements~~

~~The authors are grateful for the financial support from the National Key R&D Program of China (Grant No. 2019YFD1001602) and the Provincial Department of Science and Technology of Zhejiang, China(Grant NO.2017C02022).~~

Acknowledgments

Formatted: Font color: Auto

The authors would like to thank Changsheng Zhan (Taihu County Huayuan Agricultural Science and Technology Development Co. LTD,Anqing,Anhui, China) for the nursery management and site support.

Formatted: Font: Not Bold

Formatted: Justified, Indent: First line: 2 ch, Space Before: 0 pt, After: 0 pt, Line spacing: single

References

[1] Dai X, Sun W, Fan Q, Luo P, He J. Physicochemical property of mixed substrates with agricultural and forestry wastes and comprehensive evaluation of their effect on growth of Camellia oleifera seedlings.Journal of Plant Resources and Environment. 2016; 25(1):54-61.doi: ~~https://doi.org/~~10.3969/j.issn.1674-7895.2016.01.07

[2] Guo W, Qi W, Wang Z, Wang C. Analysis on the research status and development of agriculture and forestry waste resource utilization under the condition of blending and briquetting. Journal of China Agricultural University. 2021; 26(1): ~~(1) :~~143-150.doi: ~~https://doi.org/~~10.11841/j.issn.1007-4333.2021.01.15

[3] Zhao Y, Liu J, Zhang B. Effects of Different Substrate Ratio of Agricultural and Forestry Waste and Slow-release Fertilizer on the Growth of Ornamental Pepper. Horticulture & Seed. 2021; 41(09): ~~(09) :~~6-9. doi: ~~https://doi.org/~~10.16530/j.cnki.cn21-1574/s.2021.09.003

[4] Lu H, Chen W, Su Y, Xie P. Research on fertilizer effect of forest waste compost as seedling substrate. Rural Economy and Technology. 2021; ~~32~~32(10): ~~(10) :~~17-20.

[5] Nie L. Research on the present situation and development strategy of Agricultural waste resource recovery in China. Agricultural Economic Research. 2017; 07 ~~:~~ :59-60.

[6] Wu Q, Chen J, Gan F, Li C, Jiang H, Zheng H.Application of Well-composted Sawdust and Litterfall Substrate to Camellia oleifera Seedling. Guangxi Forestry Science. 2017; 46(4): ~~(4) :~~431-435. doi: ~~https://doi.org/~~10.19692/j.cnki.gfs.2017.04.021

[7] Luo J, Tan X, Peng Y. A Study on the Composting Technology for Camellia Shell Medium. Acta Agriculturae Universitatis Jiangxiensis. 2011; 33(4): ~~(4) :~~0712-0718.doi: ~~https://doi.org/~~10.13836/j.jjau.2011127

[8] Zhuang R. China camellia.Beijing: China Forestry Publishing House, 2008.

[9] Wang M, Zhang Y, Tang X. Report of Light Media Nursery Test For Camellia. Forestry and Environmental Science. 2018; 34(6): ~~(6) :~~56-60.

[10] Zhang X, Ding X, Zhang Y, Liu Y, Cai J, Li Y. Effects of nutrient defifi ciencies on root system in Camellia gauchowensis seedlings. Nonwood Forest Research. 2014; 32(4): ~~(4) :~~170-174. doi: ~~https://doi.org/~~10.14067/j.cnki.1003-8981.2014.04.033

[11] Wang Q, Peng G. Influence of substrate and time on cuttage steckling of Camellia oleifera. Forest By-Product and Speciallity in China. 2021; 04 ~~:~~ :001-003.doi: ~~https://doi.org/~~10.13268/j.cnki.fbsic.2021.04.001

[12] Li Y, Ma J, Wang D, Wei Y, Ye H. Effects of Mediums with Different Physicochemical Properties on Cutting Rooting of Camellia osmantha.Chinese Journal of Tropical Agriculture. 2020; ~~—~~40(3): ~~(3) :~~25-30. doi: ~~https://doi.org/~~10.12008/j.issn.1009-2196.2020.03.005

[13] Lv B, Jiang M, Ye F, Zheng X, Yu S. Research on matrix formula of oil tea container seedling. South China Agriculture. 2017; ~~—~~11(5): ~~(5) :~~24-26. doi:

https://doi.org/10.19415/j.cnki.1673-890x.2017.05.014

[14] Yang L, Chen W, Chen X, Chen J, Weng C, Zheng D. Effects of different light substrates and containers on rooting of camellia oil cuttings and evaluation of matrix bagging methods. Modern gardening. 2018; 10:43-45. doi: https://doi.org/10.14051/j.cnki.xdyy.2018.19.022

[15] Liu C, Li J, Shu Q. Growth Comparison of Camellia oleifera Seedlings Indoor and Outdoor with Different Substrates. Guangxi Forestry Science. 2019; 48(3): （3） ：332-335. doi: https://doi.org/10.19692/j.cnki.gfs.2019.03.010

[16] She Y, Zhang Y, Cao K, Li X, Zhang L, Wang Y. Effects of different culture medium formulas on roots biomass and morphology of Camellia oleifera container seedling. Non-wood Forest Research. 2020; 38(4): （4） ：11-16. doi: https://doi.org/10.14067/j.cnki.1003-8981.2020.04.002

[17] Hua M, Wang J. Experimental instruction of soil physics. Beijing: Beijing Agricultural University Press. 1993;61-73.

[18] GB/T 33891-2017， Organic substrates for greening. Beijing: Standards Press of China, 2017.(in Chinese)

[19] Shuang L, Ni S, Du J, Teng Z, Shi C. Determination of Organic Carbon in Geochemical Soil Sample by Potassium Dichromate Oxidation-Heating Method. Anhui Chemical Industry. 2016; 42(4): 110-112.

[20] Bremner JM Nitrogen-total. In: Sparks, D.L. (Ed.), Methods of Soil Analysis. Part 3 – Chemical Methods. SSSA Inc., ASA Inc., Madison, WI, USA, pp. 1996;1085-1122.

[21] Tiquia SM, Tam NFY and Hodgkiss IJ Effects of turning frequency on composting of spent pig-manure sawdust litter. Bioresource Technology 1997; 62(1-2): 37-42.

[22] Wang Y, Li M, Qiu H, Zhang W, Zhang C, Li Y. Changes of microbial quantity and nutrient content in different composting of livestock manure. Journal of Gansu Agricultural University. 2017; 52(3), 37-45. doi: https://doi.org/10.13432/j.cnki.jgsau.2017.03.007

[23] Qiao X, Shen G, Wang Z, Guo C, Liu Y, Lei Z, et al.Qiao, X, Shen, G, Wang, Z, Guo, C, Liu, Y, Lei, Z, Zhang, Z. Co-composting of livestock manure with rice straw: Characterization and establishment of maturity evaluation system. Waste management, 2014; 34(2):530-535. doi: https://doi.org/10.1016/j.wasman.2013.10.007

[24] Guardia A.D, Mallard P, Teglia C, Marin A, Pape C.L, Launay M, et al. Guardia, A.D, Mallard, P, Teglia, C, Marin, A, Pape, C.L, Launay, M, Benoist, J.C, Petiot, C. Comparison of five organic wastes regarding their behaviour during composting: part 1, biodegradability, stabilization kinetics and temperature rise. Waste Management,. 2010; 30(3), :402-414. doi: https://doi.org/10.1016/j.wasman.2009.10.019

[25] Ma R, Li D, Qi C, Li G, Wang G, Liu Y, et al. Effects of C/N ratio on compost maturity and odor emission of chicken manure. Transactions of the Chinese Society of Agricultural Engineering. 2020; 36(24): （24） ：194-202. doi: https://doi.org/10.11975/j.issn.1002-6819.2020.24.023

[26] Wang Q, Liu G, Zhang R, Li Y, Liu A, Wang W. Effects of Physical and Chemical Properties of the Substrate on the Growth of Container Seedlings of Ficus religiosa. Journal of Northwest Forestry University. 2021; 36(5): （5） ：88-93. doi: https://doi.org/10.3969/j.issn.1001-7461.2021.05.1

[27]Wu J. Comparison of physical and chemical properties of several solid cultivation substrates. Journal ofJilin Agricultural Sciences. 2006;~~–~~31(4): ~~（4）：~~17- 20.doi: ~~https://doi.org/~~10.16423/j.cnki.1003-8701.2006.04.006

[28]LYT 2314-2014.Technical specification for seed raising of oil tea containers.Beijing: State Forestry Administration, 2014. (in Chinese)

[29]Wu J, Li X, An S. Effects of Different Substrate Ratios on the Growth of Sour Pummelo Seedlings. Chinese Journal of Tropical Crops. 2018;~~-~~39(3): ~~（3）：~~443-447. doi: ~~https://doi.org/~~10.3969/j.issn.1000-2561.2018.03.006

[30]Zeng L, Li J, Wu F, Huang S, Li M, Cai Y. Effects of different substrate formulations on substrate nutrient content and seedling growth of Macadamia nut. Northern Horticultur. 2021; 24~~：~~:51-56. doi: ~~https://doi.org/~~10.11937/bfyy.2021135

[31]Chen H. Studies on factors concerned in nutrient media aeration improving growth as well as yield in cucumber. Shenyang,Liaoning, Shenyang Agricultural University, 2009.

[32]Wang Y, Shao X, Huang X, Wang K. Advances in studies on nitrogen uptake in plant roots. Pratacultural Science. 2010;~~-~~27(7): ~~（7）：~~105-111.

[33]Peng Z. Absorption, transportation and regulation of nitrogen element in plants. Journal of Hebei Agricultural University. 2019; 42(2): ~~（2）：~~1-5. doi: ~~https://doi.org/~~10.13320/j.cnki.jauh.2019.0024

[34]Ma S. Effects of Application Amount and Fertilization Modes of N,P and K on Growth,Yield and Quality of Eggplants. Shandong, Shandong agricultural University, 2018.

[35]Li C. Effects of Nitrogen,Phosphorus and Potassium Fertilization on the Growth and Physiological Characteristics of Saraca Dives Seedlings.Guangxi, Guangxi University, 2020.

[36]Yin J. Effects of Nitrogen Rate and Ratio of Nitrogen,Phosphorus,and Potassium on Dry Matter Accumulation and Grain Filling of Castor Bean (Ricinus Communis L.). Jiangsu,Yangzhou University, 2019.

[37]Lu M, Jiang F, Song X. Assessing indices of container seedling quality. Chinese Journal of Applied Ecology. 2002;~~—~~13(6): ~~（6）：~~763-765. doi: ~~https://doi.org/~~10.13287/j.1001-9332.2002.0181

[38]Ge Y, Zhu J, Jiang B, Yuan W, Shen A. Study on evaluation index of container seedling quality. Journal of Zhejiang Forestry Science and Technology. 2006; 26(1): ~~（1）：~~10-12, 22.

[39]Wang X, Lin X, Li Y, Ruan Y, Xing X. Physical and chemical properties of several kinds of agriculture and forestry waste composite matrix and their effect on container seedling of Phoebe chekiangensis. Journal of Zhejiang A & F University. 2013;~~-~~30(5): ~~（5）：~~674 - 680.

[40]Li J, Zhao X, Wei S, Zheng J, Hu Y. Study on the Physico-Chemical Properties of soil-Less Cultural Substrates of Pollution-Free Vegetable. Journal of Southwest Agricultural University. 2000;~~—~~22(2): ~~（2）：~~112-115. doi: ~~https://doi.org/~~10.13718/j.cnki.xdzk.2000.02.006

[41]Pang S, Ma Y, Zhang P, Lao Q, Yang B, Liu S. Effects of Substrate Ratio and Applying Dosages of Slow-release Fertilizer on the Growth of Container Parashrea

chinensis Seedlings. Journal of Northwest Forestry University. 2018;~~-~~33(6): ~~（6）：~~ 66-71. doi: ~~https://doi.org/~~10.3969/j.issn.1001-7461.2018.06.11

[42]Peng S, Chen Y, Yang X, Wang X, Ma L, Wang R, et al. Study on the Classification of Seedling Quality of Oil-tea Camellia (Camellia oleifera). Chinese Agricultural Science Bulletin. 2012; 28(25): ~~（25）：~~101-105.

Tables and Figures

~~[42]~~

Table 1 Primary properties of compost raw materials

Raw material/Property	TOC (%)	TN (%)	C/N	Cellulose/%	Hemicellulose/%	Lignin/%
Camellia oleifera shells	51.80±2.58	0.729±0.03	71.359±3.74	18.62±0.22	49.34±0.07	29.71±0.14
Brown silk chips	46.80±2.23	2.68±0.14	17.557±1.76	28.15±0.18	20.58±0.08	44.08±0.16
Pine chips	54.20±2.74	0.285±0.01	190.964±5.19	48.36±0.11	27.59±0.12	19.58±0.21
Chicken mature	34.39±1.75	2.73±0.14	12.667±1.31	-	-	-
Sheep mature	36.47±1.81	2.78±0.14	13.186±1.32	-	-	-
Urea	20±0.18	46.4±1.22	43.1±1.12	-	-	-

"-"indicates extremely low content or none

Table 2 Formulas of compound substrates

Treatments	Substrate formula
A	(Camellia oleifera shell + urea):Vermiculite: Perlite=6:3:1
B	(Camellia oleifera shell + sheep manure):Vermiculite: Perlite=6:3:1
C	(Camellia oleifera shell + palm fiber residue+chicken manure):Vermiculite: Perlite=6:3:1
D	(Camellia oleifera shell + palm fiber residue+chicken manure):Boiler slag:Vermiculite: Perlite=4:2:3:1
E	(Pine chip + urea):Vermiculite: Perlite=6:3:1

Table 3 Physical and chemical properties of the composts of agricultural and forest waste and other components for the preparation of compound substrates

Group	Moisture content MC/%	bulk density g·cm^{-3}	Total porosity %	Aeration porosity %	Water-holding porosity %	pH	EC (ms·cm^{-1})
A1	10.27±0.52	0.39±0.018	73.94±3.68	6.07±0.29	67.87±3.39	7.00±0.21	0.13±0.02
A2	9.46±0.48	0.40±0.021	74.13±3.70	9.30±0.47	64.83±3.23	8.00±0.26	0.08±0.03
A3	13.82±0.68	0.47±0.024	65.90±3.31	11.83±0.60	54.07±2.71	7.79±0.24	0.20±0.01
A4	22.47±1.11	0.42±0.02	83.40±4.16	1.80±0.089	81.60±4.07	7.00±0.22	0.06±0.03

Group	TN%	TK%	TP%	TOC%	total nutrient %	GI%	organic matter %
A1	1.07±0.05	1.83±0.09	0.37±0.02	38.20±1.92	3.27±0.15	89.12±4.44	65.86±3.25
A2	1.16±0.057	2.07±0.12	0.62±0.03	36.30±1.82	3.85±0.21	90.13±4.49	62.58±3.11
A3	2.75±0.14	2.55±0.11	1.02±0.05	29.80±1.48	6.32±0.33	99.42±4.98	51.38±2.54
A4	0.84±0.04	0.54±0.03	0.42±0.02	47.30±2.37	1.80±0.08	91.31±4.52	81.55±4.06

Raw	Moisture content	bulk density g·cm	Total porosity %	Aeration	Water-holding	pH	EC(ms·cm^{-1})

Formatted: No bullets or numbering, Tab stops: Not at 0.22"

Formatted: Font: Bold

Formatted: Font:

Formatted: Centered, Indent: First line: 0", Space After: 0 pt

Formatted: Font:

Formatted: Centered, Indent: First line: 0", Space After: 0 pt

materials	MC/%	3		porosity %	porosity %		
Boiler slag	2.08±0.11	0.70±0.03	63.10±3.16	0.40±0.019	62.7±3.14	8.00±0.025	0.22±0.012
Vermiculite	1.55±0.078	0.13±0.01	95.04±4.75	30.03±1.51	65.01±3.24	7.00±0.021	0.047±0.0024
perlite	0.14±0.007	0.16±0.01	60.33±3.03	29.51±1.48	30.82±1.55	7.00±0.019	0.022±0.0011

Table 4 Changes in the physical and chemical properties of compound substrates after seedling cultivation

group		A	B	C	D	E
bulk density g/cm^3	before	0.41±0.02	0.47±0.02	0.41±0.02	0.42±0.02	0.41±0.02
	after	0.57±0.027	0.76±0.037	0.59±0.028	0.59±0.029	0.60±0.031
	increase	39.02±4.39	61.70±2.77	43.90±2.17	40.48±2.02	46.34±2.20
total porosity %	before	73.83±3.65	67.83±3.34	66.70±3.37	68.17±3.37	74.60±3.68
	after	58.07±2.86	58.80±2.94	61.40±3.17	64.50±3.29	59.60±3.95
	decrease	21.35±1.03	13.31±0.63	7.95±0.36	5.38±0.22	20.11±0.95
aeration porosity %	before	29.31±1.35	28.12±1.31	25.44±1.22	24.22±1.19	23.10±1.12
	after	52.11±2.33	54.21±2.34	51.91±2.19	44.22±2.16	42.18±2.02
	increase	77.79±3.82	92.78±4.55	104.05±5.07	82.58±3.92	82.60±3.85
Water-holding porosity %	before	44.52±2.21	39.71±1.94	41.26±1.99	43.95±2.08	51.50±2.42
	after	5.96±0.22	4.60±0.21	9.49±0.42	8.30±0.37	17.41±0.83
	decrease	86.61±4.25	88.42±4.31	77.00±3.68	81.11±3.94	66.19±3.20
pH	before	7.43±0.29	7.38±0.24	7.03±0.26	7.34±0.27	7.20±0.23
	after	7.32±0.34	7.45±0.36	7.65±0.37	7.48±0.36	7.61±0.37
	increase%	-1.48±0.067	0.95±0.041	8.82±0.41	1.91±0.094	5.69±0.26
EC ($ms \cdot cm^{-1}$)	before	0.15±0.002	0.15±0.002	0.20±0.002	0.12±0.003	0.12±0.003
	after	0.46±0.023	0.22±0.012	0.24±0.007	0.18±0.005	0.24±0.002
	increase%	206.66±2.0	46.00±2.3	20.00±1.4	50.00±2.3	50.00±1.33

Table 5 Changes in the nutrient contents of compound substrates after seedling cultivation

group		A	B	C	D	E
organic matters %	before	54.33±2.72	50.90±2.53	43.00±2.13	37.00±1.82	61.50±3.06
	after	9.45±0.46	19.14±0.94	14.43±0.73	9.93±0.48	15.76±0.77
	decrease	82.61±5.85	62.40±6.82	66.44±6.65	73.16±6.22	74.37±3.51
N%	before	0.86±0.041	0.93±0.042	2.29±0.09	1.38±0.065	0.85±0.039
	after	0.49±0.022	0.51±0.023	0.68±0.032	0.65±0.031	0.24±0.011
	decrease	43.02±1.98	45.16±2.04	70.31±3.32	52.90±2.39	71.76±3.41
P_2O_5%	before	0.36±0.017	0.56±0.026	0.90±0.042	0.59±0.027	0.62±0.029
	after	0.095±0.0045	0.16±0.0079	0.58±0.025	0.22±0.0099	0.069±0.0032
	decrease	73.61±3.33	71.43±3.21	35.56±1.56	62.71±2.71	88.87±4.03
K_2O%	before	1.47±0.074	1.68±0.082	2.13±0.097	1.43±0.068	0.75±0.035
	after	0.59±0.027	0.72±0.034	0.63±0.031	0.61±0.029	0.43±0.021
	decrease	59.86±2.86	57.14±2.74	70.42±3.43	57.34±2.73	42.67±1.87
Total nutrient %	before	2.69±0.12	3.17±0.14	5.32±0.25	3.40±0.18	2.22±0.12
	after	1.18±0.06	1.40±0.07	1.89±0.09	1.47±0.07	0.75±0.03
	decrease	56.13±6.32	55.84±6.62	64.47±6.39	56.76±7.35	66.22±7.21

Formatted: Line spacing: single

Table 6 Morphologies of seedlings raised on different substrates

**Treatment**		**A**	**B**	**C**	**D**	**E**
survival rate %		99	99.5	100	97.5	99.5
height/cm	average	20.0±0.95	27.2±1.33	31.4±1.55	29.6±1.47	22.2±1.08
	Maximum	44.0±2.10	51.0±2.46	63.0±3.05	47.0±2.23	38.0±1.85
Ground diameter/mm	average	2.8±0.13	3.7±0.17	4.1±0.22	3.9±0.18	3.3±0.15
	Maximum	3.9±0.18	4.7±0.22	5.5±0.26	5.1±0.23	4.4±0.21
total root length/cm		2169.79±107.96	4377.77±215.32	2161.92±107.21	2864.55±142.53	3900.97±192.67
Root surface area/cm^2		371.30±18.23	768.81±38.23	397.98±19.56	596.43±29.62	683.89±34.01
Average root diameter/mm		0.56±0.026	0.56±0.027	0.58±0.027	0.66±0.032	0.56±0.025
Root volume cm^3		5.10±0.24	10.86±0.52	5.90±0.30	10.01±0.51	9.58±0.45

Table 7 Pearson correlation analysis of physical and chemical properties of substrate and seedling morphology

	**Ground diameter**	**Seedling height**	**Total root length**	**Root surface area**	**Moisture content**	**Total porosity before seedling cultivation**	**Total aeration porosity before seedling cultivation**	**Organic matter content before seedling cultivation**	**Total nutrient content before seedling cultivation**	**Total nutrient after seedling cultivation**	**Change in organic matter content**	**Change in total nutrient content**
Ground diameter	1	0.982**	0.044	0.181	-0.972**	-0.891*	-0.438	-0.723	0.754	0.723	-0.905*	0.732
Seedling height		1	-0.094	0.049	-0.997**	-0.944*	-0.311	-0.823	0.820	0.830	-0.957*	0.773
Total root length			1	0.971**	0.080	0.059	-0.166	0.412	-0.497	-0.471	0.087	-0.486
Root surface area				1	-0.055	-0.055	-0.282	0.219	-0.447	-0.393	-0.074	-0.452
Moisture content					1	0.967**	0.238	0.818	-0.826	-0.853	0.966**	-0.771
Total porosity before seedling cultivation						1	-0.005	0.100	-0.039	0.176	0.130	-0.148
Total aeration porosity before seedling cultivation							1	0.838	-0.814	-0.937*	0.952*	-0.710
Organic matter content before seedling cultivation								1	-0.669	-0.810	0.906*	-0.562
Total nutrient content before seedling cultivation									1	0.935*	-0.728	0.983**
Total nutrient after seedling cultivation										1	-0.841	0.855
Change in organic matter content											1	-0.633
Change in total nutrient content												1

*- Correlated at the significant level of 0.05 (bilateral)

**-Correlated at the significant level of 0.01(bilateral)

Figure 1 Temperature changes of the four compost piles during composting

Formatted: Centered, Indent: First line: 0", Space After: 0 pt

Formatted: Centered, Indent: First line: 0", Space After: 0 pt

Formatted: Font: 8 pt, Bold

Formatted: Space After: 0 pt, Line spacing: single

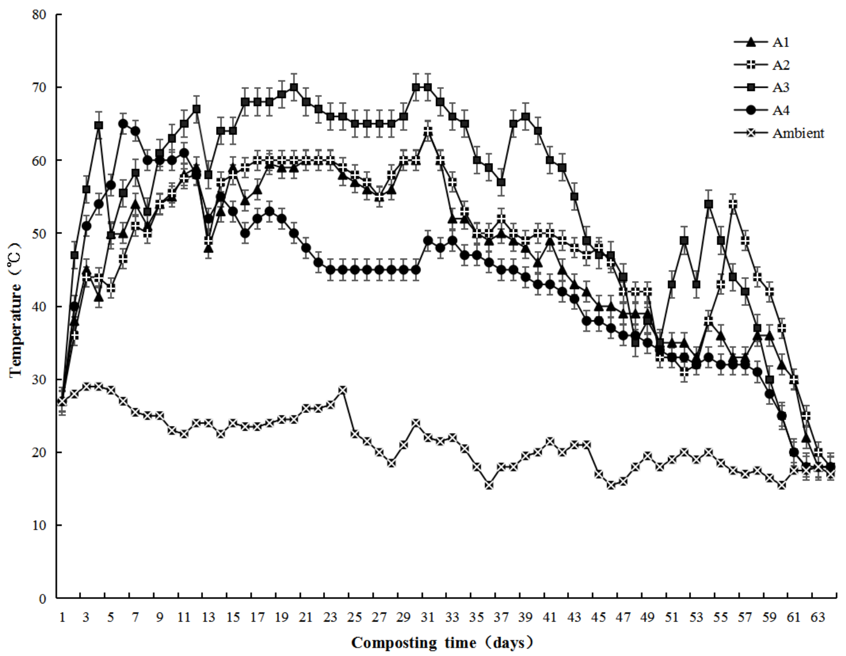

Formatted: Centered, Indent: First line: 0", Space After: 0 pt

Dear Felix Yao Huemabu Kutsanedzie, Ph.D,

I have read your reply to the manuscript and modified it according to your opinions. The following are the replies one by one.

The whole manuscript has been numbered.

The format of the references has been revised to meet plos ONE requirements.

The grammar of the whole article has been checked and revised in detail, including "we" and "the paper or study....".

The format of tables and figures in the article was also modified, and the tables and pictures were all put at the end of the manuscript. Only "Figure 1" and "Table 1" were left in the position of the charts in the text for illustration.

Sincerely yours,

Jinping Zhang
