# Supplementary material for: Formulation of substrates with agricultural and forestry wastes for Camellia oleifera Abel seedling cultivation
Source: PLoS One. 2022 Jul 18;17(7):e0265979. doi: 10.1371/journal.pone.0265979 (PMC9292103; doi:10.1371/journal.pone.0265979)
Supplement: Attachment — Submitted filename: Response to Reviewers.docx [file pone.0265979.s002.docx]

Dear Felix Yao Huemabu Kutsanedzie, Ph.D,

I have read your reply to the manuscript and modified it according to your opinions. The following are the replies one by one.

The whole manuscript has been numbered.

The format of the references has been revised to meet plos ONE requirements.

The grammar of the whole article has been checked and revised in detail, including "we" and "the paper or study....".

The format of tables and figures in the article was also modified, and the tables and pictures were all put at the end of the manuscript. Only "Figure 1" and "Table 1" were left in the position of the charts in the text for illustration.

Sincerely yours,

　　Jinping Zhang
