# Supplementary material for: Formulation of substrates with agricultural and forestry wastes for Camellia oleifera Abel seedling cultivation
Source: PLoS One. 2022 Jul 18;17(7):e0265979. doi: 10.1371/journal.pone.0265979 (PMC9292103; doi:10.1371/journal.pone.0265979)
Supplement: Attachment — Submitted filename: Response to Reviewers.docx [file pone.0265979.s004.docx]

Dear Solna Carreon Santos,

I have read your reply to the manuscript and submitted the minimum data set according to your opinion. The following are some minor problems that we found and revised when we summarized the data and made the final accounting, which will be explained here.

In the process of writing the paper, there were some manual errors in the data processing, for example, there were several misread and input errors in the data input, and the standard deviation of part of the mean (fluctuation range) was substituted incorrectly in the calculation, leading to a series of errors in the relevant standard deviation. Fortunately, these errors did not affect the analysis in our paper, and we found them and corrected them one by one before the paper was finalized.

Sincerely yours,

　　Jinping Zhang
